# Screening prevalence of fetal alcohol spectrum disorders in a region of the United Kingdom: A population-based birth-cohort study

**DOI:** 10.1016/j.ypmed.2018.10.013

**Published:** 2019-01

**Authors:** Cheryl McQuire, Raja Mukherjee, Lisa Hurt, Andrea Higgins, Giles Greene, Daniel Farewell, Alison Kemp, Shantini Paranjothy

**Affiliations:** a Bristol Population Health Science Institute, University of Bristol, Bristol, UK; b Surrey and Borders Partnership NHS Foundation Trust, Redhill, UK; c Division of Population Medicine, Cardiff University, Cardiff, UK; d School of Psychology, Cardiff University, Cardiff, UK

**Keywords:** ALSPAC, Avon Longitudinal Study of Parents and Children, ARND, alcohol related neurodevelopmental disorder, CNS, central nervous system, FAS, fetal alcohol syndrome, FASD, fetal alcohol spectrum disorders, PAE, prenatal alcohol exposure, pFAS, partial fetal alcohol syndrome, Fetal Alcohol Spectrum Disorders, Prevalence, Epidemiology, Alcohol, Pregnancy, Developmental disability, ALSPAC

## Abstract

Fetal alcohol spectrum disorders (FASDs) are lifelong disabilities caused by prenatal alcohol exposure. Prenatal alcohol use is common in the UK, but FASD prevalence was unknown. Prevalence estimates are essential for informing FASD prevention, identification and support.

We applied novel screening algorithms to existing data to estimate the screening prevalence of FASD. Data were from a population-based cohort study (ALSPAC), which recruited pregnant women with expected delivery dates between 1991 and 1992 from the Bristol area of the UK. We evaluated different missing data strategies by comparing results from complete case, single imputation (which assumed that missing data indicated no exposure and no impairment), and multiple imputation methods.

6.0% of children screened positive for FASD in the analysis that used the single imputation method (total N = 13,495), 7.2% in complete case analysis (total N = 223) and 17.0% in the analysis with multiply imputed data (total N = 13,495). A positive FASD screen was more common among children of lower socioeconomic status and children from unplanned pregnancies. Our analyses showed that the complete case and single imputation methods that are commonly used in FASD prevalence studies are likely to underestimate FASD prevalence.

Although not equivalent to a formal diagnosis, these screening prevalence estimates suggest that FASD is likely to be a significant public health concern in the UK. Given current patterns of alcohol consumption and recent changes in prenatal guidance, active case ascertainment studies are urgently needed to further clarify the current epidemiology of FASD in the general population of the UK.

## Highlights

• Up to 79% of women consumed alcohol while pregnant.
• Up to 17% of children screened positive for a fetal alcohol spectrum disorder (FASD).
• Alcohol-related neurodevelopmental disorder was the most common FASD subtype.
• Screening is not equivalent to formal diagnosis. Follow-up studies required.
• A positive FASD screen was more common following unplanned pregnancy.

## 1 Introduction

Prenatal alcohol use can lead to lifelong disabilities, known as fetal alcohol spectrum disorders (FASDs) (British Medical Association (BMA), 2016). FASD is an umbrella term that describes a range of features including facial dysmorphia, growth deficiency and neurobehavioural impairment. It is associated with over 400 comorbid conditions and adverse outcomes in later life (Popova et al., 2016; Streissguth et al., 2004). FASD is a leading cause of developmental disability. Studies from the USA and Europe suggest that 1% to 10% of children in the general population have FASD (Lange et al., 2017a; Roozen et al., 2016; May et al., 2018). In rural South Africa, up to 28% of children have FASD (May et al., 2017).

Despite having the fourth highest estimated prevalence of prenatal alcohol use worldwide (Popova et al., 2017), the prevalence of FASD in the UK is unknown. In 2015/16, the All Party Parliamentary Group for FASD and British Medical Association expressed an urgent need for a UK population-based prevalence study to guide FASD prevention efforts and policy for alcohol use in pregnancy (British Medical Association (BMA), 2016; All Party Parliamentary Group on FASD, 2015). Active case ascertainment methods, such as in-school screening methods, are the preferred approach for FASD prevalence studies; however, they are costly and resource intensive (May et al., 2009). To date, proposals to conduct active case ascertainment studies of FASD in the UK have not been successful (All Party Parliamentary Group on FASD, 2015). To address this knowledge gap, we developed novel FASD screening algorithms and applied these to existing data from a population-based birth-cohort in England to estimate FASD screening prevalence. We also investigated the impact of using different missing data strategies when estimating FASD prevalence.

## 2 Participants and methods

### 2.1 Data source

We used data from the Avon Longitudinal Study of Parents and Children (ALSPAC) cohort, a prospective population-based birth-cohort study that recruited 14,541 pregnant women with expected delivery dates between 1st April 1991 to 31st December 1992 from the Bristol area of the UK (Boyd et al., 2013; Fraser et al., 2013). The ALSPAC cohort includes extensive repeated measures of prenatal exposures, developmental outcomes and sociodemographic factors, collected from questionnaires, in-clinic assessments and data linkage. ALSPAC sample characteristics, methodology and representativeness are described in previous publications (Boyd et al., 2013; Fraser et al., 2013) and online (http://www.alspac.bris.ac.uk/welcome/index.shtml). The study website contains details of all data (http://www.bris.ac.uk/alspac/researchers/data-access/data-dictionary/).

### 2.2 Study approval

Ethical approval was obtained from the ALSPAC Ethics and Law Committee (IRB00003312) and the Local Research Ethics Committees (Avon Longitudinal Study of Parents and Children (ALSPAC), 2018) and study approval was granted by the ALSPAC Executive Committee on March 2nd, 2016 (Project B2620).

### 2.3 Participants

We included data on all singleton pregnancies in the core ALSPAC sample. We excluded children who were not alive at one year of age, those with genetic conditions, and those who did not speak English as a primary language. Participants who were in the armed forces social class category were excluded due to sparse data, which led to an inability to reach convergence in imputation models.

### 2.4 FASD screening algorithm development and validation

We used the FASD Canadian guidelines for diagnosis (2005) (Chudley et al., 2005) to develop FASD screening algorithms. A detailed description of algorithm development and validation is provided elsewhere (McQuire, 2018). Appendix 1 provides full algorithm specifications. First, we identified ALSPAC measures relevant to the Canadian FASD criteria. Second, we derived a series of algorithm specifications that corresponded to different combinations of central nervous system (CNS) and prenatal alcohol exposure (PAE) criteria. Specifications for the CNS criteria ranged from what we referred to as ‘Liberal’, ‘Mid’ and ‘Strict’ criteria, corresponding to increasing levels of convergent evidence and symptom severity. Following the case conference validation process (described below), we added a ‘Revised’ CNS category to reflect modifications to the CNS criteria, following recommendations from the panel. Similarly, the PAE specifications ranged from what we termed ‘Any’, ‘Mid’ and ‘Strict’, corresponding to increasing levels of exposure (dose and/or duration). Any PAE was defined as any level of prenatal alcohol exposure at any time in pregnancy; Mid PAE was defined as two trimesters of prenatal alcohol exposure and/or binge drinking and Strict PAE was defined as three trimesters of prenatal alcohol exposure and/or binge drinking. We also tested other thresholds for PAE that have been suggested in the literature (see Appendix 1). Third, we selected a stratified random sample of 31 participant profiles to be considered by an expert case-conference panel (see Appendix 2 for full details of the case-conference sampling strategy). The expert panel included a consultant psychiatrist from the UK National Clinic for FASD (RM), a paediatrician (AK), and an educational psychologist (AH). The panel were given the participant profiles and asked to decide whether, on the balance of probability, a diagnosis of FASD would be made in clinic, given the information provided. Panel members were blind to the FASD classification status that had been assigned by the algorithms. Decisions were reached by consensus. We selected the algorithms with the greatest levels of agreement with the expert panel for prevalence analyses.

### 2.5 Outcome

**Fig. 1:**
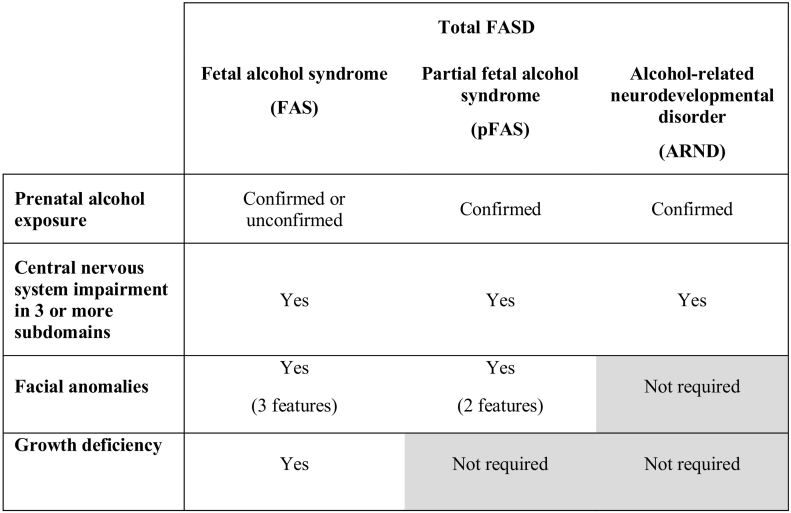
Summary of FASD subtypes and core features.

The primary outcome was total FASD screening prevalence, defined as the proportion of participants who met criteria for any condition within the FASD continuum, based on the FASD screening algorithm. Secondary outcomes were the prevalence of FASD subtypes (described in Fig. 1).

It is important to note that FASD diagnosis requires input from a multidisciplinary team, with an opportunity to interact with the child and their caregivers, to allow a thorough analysis of a child's developmental profile and consider differential diagnoses. For the purposes of this research, ‘cases’ and ‘participants with FASD’ refer to children who met the screening algorithm criteria for FASD. This is not equivalent to a formal FASD diagnosis.

### 2.6 Statistical analyses

Analyses were conducted in Stata 14.2 (Stata Statistical Software [computer program]. Release 14, 2015).

#### 2.6.1 Algorithm validation

We calculated diagnostic accuracy statistics to quantify the level of agreement between the FASD classifications that were made by the expert panel and the algorithms. Algorithm performance was quantified using sensitivity and specificity statistics and the 0,1 method, which identifies the shortest distance to the top left-hand corner of a receiver operating characteristic plot (Kelly et al., 2008). Lower values of the 0,1 statistic indicate better performance.

#### 2.6.2 Missing data methods

To address the bias and imprecision introduced by missing data, and to evaluate the impact of different missing data strategies, we produced prevalence estimates using data from complete case, single and multiple imputation methods. We compared patterns of PAE and clinical characteristics across each of the missing data strategies to investigate how this influenced prevalence estimates (presented in Appendix 3).

In complete case analyses, we excluded all children who had missing data on any of the measures that were included in the FASD screening algorithm. In the single imputation method, we assumed that missing PAE data indicated no exposure and that missing phenotype data indicated no impairment. The multiple imputation model specification (Royston, 2009) and missing data frequencies are presented in Appendix 4.

### 2.7 Prevalence estimation

We generated prevalence estimates for total FASD and FASD subtypes (fetal alcohol syndrome [FAS], partial fetal alcohol syndrome [pFAS] and alcohol-related neurodevelopmental disorder [ARND]) by applying the FASD screening algorithms to the dataset. Total FASD prevalence was defined as the number of participants in the eligible sample who met criteria for any FASD subcategory, divided by the total eligible sample. To ensure compliance with ALSPAC policy, we combined prevalence estimates for the less common FASD subtypes (pFAS and FAS) if fewer than five participants met criteria for one of these categories and censored estimates when fewer than five participants met criteria for the combined pFAS/FAS subcategory. We used the Wilson method to generate confidence intervals for complete case and singly imputed data (Newcombe, 1998). We used Rubin's combination rules to derive prevalence estimates and confidence intervals for multiply imputed data (Little & Rubin, 2002).

## 3 Results

### 3.1 Participants

**Fig. 2:**
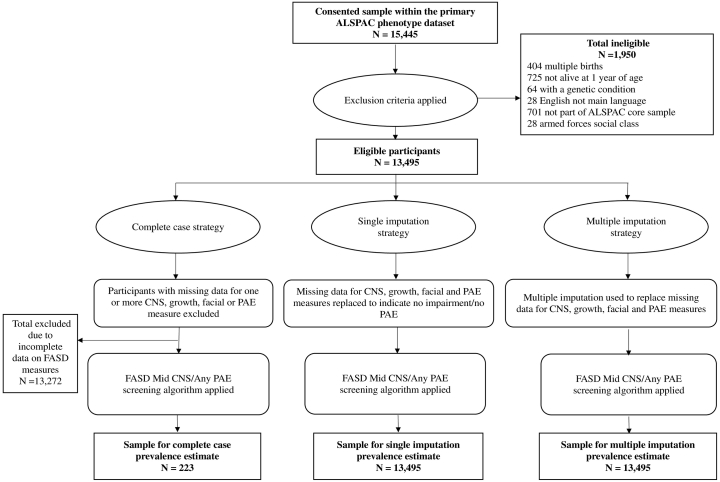
Flow diagram of the eligible and final sample for the primary FASD prevalence estimates, by missing data strategy.

Fig. 2 provides a participant flow diagram, by imputation strategy. There were 15,445 consented children in the ALSPAC dataset and, of these, 13,495 were eligible for inclusion. This sample size was preserved using the single and multiple imputation missing data strategies. Missing data led to a substantial reduction in the size of the complete case sample (N = 223). Appendix 5 provides a comparison of participants with complete versus incomplete data.

#### 3.1.1 Missing data patterns

The proportion of missing data ranged from 0% for maternal age, gestational age at delivery, and sex of the child to 70% for teacher-reported communication problems. Forty-nine percent of participants had incomplete PAE data. Participants with complete data differed from those with incomplete data on a range of characteristics, indicating that data were not missing completely at random (Appendix 5). Compared to those with complete data, mothers of children with incomplete data were younger, were more likely to report that pregnancy was unplanned, and were of lower socioeconomic status. During pregnancy, mothers of children with incomplete data were less likely to report drinking alcohol overall, but more likely to report binge drinking. They were more likely to have smoked, and to have had significant depression and anxiety symptoms. Children with incomplete data had poorer outcomes including lower IQ, conduct problems, and growth deficiency.

### 3.2 FASD screening algorithm performance

Performance results for all algorithms are shown in Appendix 6. The ‘Mid CNS/Any PAE’ algorithm had the highest performance (0,1 value = 0.46; sensitivity 91%; specificity 55%). We selected this algorithm to screen for FASD cases in our primary prevalence analyses.

To investigate the impact of applying different algorithms to the data, we selected the two algorithms with the next best values of the 0,1 statistic to be used in sensitivity analyses. These were the ‘Mid CNS/Mid PAE’ algorithm and ‘Revised CNS/Any PAE’ algorithms (both had 0,1 value = 0.47; sensitivity 64%; specificity 70%).

### 3.3 FASD screening prevalence

#### 3.3.1 Complete case prevalence estimates

Based on the complete case sample (N = 223), 7.2% (95% CI 4.5%–11.3%) of children screened positive for FASD. We do not report estimates for FASD subcategories as fewer than five participants met criteria for pFAS/FAS.

#### 3.3.2 Single imputation prevalence estimates

Using the singly imputed data (N = 13,495), 6.0% (95% CI 5.7%–6.5%) of children met criteria for FASD. ARND accounted for 5.8% (95% CI 5.5%–6.2%) of FASD cases and 0.2% (95% CI 0.1%–0.3%) met criteria for pFAS/FAS.

#### 3.3.3 Multiple imputation prevalence estimates

In analyses with multiply imputed data (N = 13,495), 17.0% (95% CI 16.1%–17.8%) of children met criteria for FASD. 15.4% (95% 14.4%–16.4%) met criteria for ARND and 1.6% (95% CI 1.1%–2.1%) met criteria for pFAS/FAS.

##### 3.3.3.1 Sensitivity analyses

Using a screening algorithm with the same CNS criteria as the primary analyses, but more stringent PAE criteria (the ‘Mid CNS/Mid PAE’ algorithm), we obtained a prevalence of 12.7% (95% CI 11.9%–13.4%) for FASD. Using an algorithm with the same PAE criteria as the primary analyses, but different CNS criteria (the ‘Revised CNS/Any PAE’ algorithm), we obtained a prevalence of 12.8% (95% CI 12.0%–13.5%) for FASD.

### 3.4 Participant characteristics

Table 1 presents sociodemographic and pregnancy characteristics and Table 2 presents PAE and clinical characteristics of the sample by FASD status, using multiply imputed data. Seventy-nine percent of mothers in the sample consumed alcohol during pregnancy. FASD was more common among children whose mothers were of lower socioeconomic status. Children with FASD were more likely to be male and to be born to mothers who reported that pregnancy was unplanned.

**Table 1:** Sociodemographic and pregnancy characteristics of participants by FASD status, based on multiply imputed data. Data are from the ongoing Avon Longitudinal Study of Parents and Children, England (core recruitment involved pregnant women with expected delivery dates between 1991 and 1992).

	Total sample N = 13,495 %^a^ (95% CI)	Not FASD N = 11,201^a^, ^b^ %^a^ (95% CI)	FASD N = 2,294^a^, ^b^ %^a^ (95% CI)
Sociodemographic factors			
Sex of the child			
Female	48·5 (47.6–49.3)	51.5 (50.5–52.4)	33.9 (31.5–36.2)
Male	51.5 (50.7–52.4)	48.5 (47.6–49.5)	66.1 (63.8–68.5)
Maternal ethnicity			
White	97.1 (96.8–97.5)	97.3 (96.9–97.6)	96.5 (95.5–97.6)
Non-White	2.9 (2.5–3.2)	2.7 (2.4–3.1)	3.5 (2.4–4.6)
Maternal age at pregnancy (years)			
<20	4.7 (4.4–5.1)	4.2 (3.8–4.7)	7.2 (5.9–8.6)
20–29	58.1 (57.3–58.9)	57.4 (56.4–58.3)	61.5 (59.3–63.8)
30+	37.2 (36.3–38.0)	38.4 (37.5–39.3)	31.2 (29.2–33.3)
Home ownership			
Mortgaged/owned	72.4 (71.6–73.2)	75.6 (74.6–76.5)	57.0 (54.3–59.6)
Council/housing association	16.6 (15.9–17.3)	14.1 (13.3–14.8)	29.0 (26.7–31.3)
Rented (private)	7.4 (6.9–7.8)	6.9 (6.4–7.4)	9.6 (8.0–11.3)
Other	3.7 (3.3–4.0)	3.5 (3.1–3.9)	4.4 (3.3–5.5)
Maternal social class			
Professional	5.0 (4.6–5.4)	5.6 (5.1–6.1)	2.1 (1.4–2.8)
Managerial/technical	28.4 (27.5–29.3)	29.6 (28.7–30.6)	22.3 (20.3–24.3)
Skilled non-manual	42.9 (41.9–43.8)	43.0 (42.0–44.0)	42.2 (39.8–44.6)
Skilled manual	8.8 (8.2–9.4)	8.3 (7.7–9.0)	11.1 (9.5–12.6)
Partly skilled/unskilled	15.0 (14.2–15.7)	13.5 (12.6–14.3)	22.3 (20.0–24.6)
Paternal social class			
Professional	9.8 (9.3–10.4)	10.8 (10.1–11.4)	5.3 (4.1–6.5)
Managerial/technical	31.6 (30.8–32.5)	32.9 (31.8–33.9)	25.6 (23.4–27.9)
Skilled non-manual	10.7 (10.1–11.3)	11.0 (10.4–11.6)	9.2 (7.6–10.7)
Skilled manual	32.8 (31.9–33.7)	31.8 (30.8–32.8)	37.4 (35.2–39.6)
Partly skilled/unskilled	15.1 (14.3–15.8)	13.5 (12.8–14.3)	22.5 (20.2–24.8)
Marital status			
Not married	26.0 (25.2–26.7)	23.8 (23.0–24.7)	36.6 (34.3–38.8)
Married	74.0 (73.3–74.8)	76.2 (75.3–77.0)	63.4 (61.2–65.7)

Pregnancy factors			
Parity			
0	44.9 (44.0–45.7)	45.7 (44.7–46.7)	40.9 (38.5–43.3)
1	34.9 (34.0–35.7)	35.0 (34.1–35.9)	34.3 (31.8–36.7)
2	14.3 (13.7–14.9)	13.9 (13.2–14.5)	16.2 (14.6–17.9)
>2	6.0 (5.6–6.4)	5.5 (5.0–5.9)	8.6 (7.1–10.1)
Preterm delivery (<37 weeks)			
Yes	5.0 (4.6–5.3)	4.6 (4.2–5.0)	6.8 (5.7–8.0)
No	95.0 (94.7–95.4)	95.4 (95.0–95.8)	93.2 (92.0–94.3)
Unplanned pregnancy			
Yes	31.4 (30.6–32.3)	29.8 (28.9–30.7)	39.3 (36.8–41.8)
No	68.6 (67.8–69.4)	70.2 (69.3–71.1)	60.7 (58.2–63.3)

a Estimates pooled across imputation sets. Estimates vary for each imputation set.

b FASD status based on the ‘Mid CNS/Any PAE’ screening algorithm. Abbreviations: CI, confidence interval; CSE, Certificate of Secondary Education; FASD, fetal alcohol spectrum disorders; N, sample size. Note: Some percentages may not add to 100% due to rounding.

**Table 2:** Prenatal alcohol exposure and clinical characteristics by FASD status based on multiply imputed data. Data are from the ongoing Avon Longitudinal Study of Parents and Children, England (core recruitment involved pregnant women with expected delivery dates between 1991-1992).

	Total sample N = 13,495 %^a^ (95% CI)	Not FASD N = 11,201^a^, ^b^ %^a^ (95% CI)	FASD N = 2,294^a^, ^b^ %^a^ (95% CI)
PRENATAL ALCOHOL EXPOSURE			
Prenatal alcohol exposure (any)			
No	21·3 (20·5 - 22·0)	25·6 (24·8 - 26·4)	Not applicable^c^
Yes	78·7 (78·0 - 79·5)	74·4 (73·6 - 75.2)	
Prenatal binge drinking			
No	74·7 (73·7 - 75·6)	77·2 (76·2 - 78·3)	62·2 (59·4 - 64·9)
Yes	25·3 (24·4 - 26·3)	22·8 (21·7 - 23·8)	37·8 (35·1 - 40·6)
Prenatal alcohol exposure (max dose/frequency per week during pregnancy)			
None^d^	32·4 (31·6 - 33·2)	35·2 (34·3 - 36·1)	19·0 (16·8 - 21·1)
<1 glass per week	42·2 (41·3 - 43·0)	40·4 (39·4 - 41·3)	51·1 (48·7 - 53·5)
1-6 glasses per week	22·1 (21·4 - 22·8)	21·5 (20·7 - 22·3)	25·1 (22·9 - 27·3)
7+ glasses per week	3·3 (3·0 - 3·6)	3·0 (2·6 - 3·3)	4·8 (3·9 - 5·8)

FACIAL PHENOTYPE			
FAS facial phenotype			
No	99·5 (99·3 - 99·7)	99·5 (99·4 - 99·7)	99·3 (98·7 - 99·9)
Yes	0·5 (0·3 - 0·7)	0·5 (0·3 - 0·6)	0·7 (0·1 - 1·3)
Partial FAS facial phenotype			
No	91·5 (90·4 - 92·7)	91·7 (90·7 - 92·7)	90·5 (87·6 - 93·5)
Yes	8·5 (7·3 - 9·6)	8·3 (7·3 - 9·3)	9·5 (6·5 - 12·4)

GROWTH			
Growth impairment (< 9^th^ percentile)			
No	91·7 (91·3 - 92·2)	92·5 (91·9 - 93·0)	88·3 (86·6 - 90·0)
Yes	8·3 (7·8 - 8·7)	7·6 (7·0 - 8·1)	11·7 (10·0 - 13·4)

CENTRAL NERVOUS SYSTEM			
CNS impairment in ≥ 3 domains			
No	78·3 (77·3 - 79·2)	94·3 (93·7 - 94·8)	Not applicable^e^
Yes	21·7 (20·8 - 22·7)	5·7 (5·2 - 6·3)	
Impaired CNS domain a) Hard and soft neurologic signs			
No	93·0 (92·2 - 93·8)	95·8 (95·2 - 96·4)	79·2 (76·4 - 82·0)
Yes	7·0 (6·2 - 7·8)	4·2 (3·6 - 4·8)	20·8 (18·0 - 23·6)
Impaired CNS domain b) Brain structure			
No	99·4 (99·1 - 99·6)	99·6 (99·4 - 99·7)	98·4 (97·5 - 99·2)
Yes	0·6 (0·4 - 0·9)	0·4 (0·3 - 0·6)	1·6 (0·8 - 2·5)
Impaired CNS domain c) Cognition			
No	44·1 (43·0 - 45·2)	48·4 (47·2 - 49·5)	23·2 (20·8 - 25·5)
Yes	55·9 (54·8 - 57·0)	51·6 (50·5 - 52·8)	76·8 (74·5 - 79·2)
Impaired CNS domain d) Communication			
No	97·0 (96·4 - 97·6)	98·7 (98·3 - 99·0)	89·0 (86·4 - 91·5)
Yes	3·0 (2·4 - 3·6)	1·3 (1·0 - 1·7)	11·0 (8·5 - 13·6)
Impaired CNS domain e) Education			
No	75·0 (74·3 - 75·8)	85·1 (84·2 - 85·9)	26·1 (23·8 - 28·3)
Yes	25·0 (24·2 - 25·8)	14·9 (14·1 - 15·8)	73·9 (71·7 - 76·2)
Impaired CNS domain f) Memory			
No	91·1 (90·0 - 92·2)	94·7 (94·0 - 95·4)	73·7 (69·7 - 77·7)
Yes	8·9 (7·8 - 10·0)	5·3 (4·6 - 6·0)	26·3 (22·3 - 30·3)
Impaired CNS domain g) Executive functioning			
No	96·1 (95·5 - 96·7)	98·1 (97·7 - 98·5)	86·1 (83·6 - 88·7)
Yes	3·9 (3·3 - 4·5)	1·9 (1·5 - 2·3)	13·9 (11·3 - 16·4)
Impaired CNS domain h) Attention deficit/hyperactivity			
No	81·7 (81·0 - 82·5)	90·3 (89·7 - 91·0)	39·7 (37·1 - 42·3)
Yes	18·3 (17·5 - 19·0)	9·7 (9·0 - 10·3)	60·3 (57·7 - 62·9)
Impaired CNS domain i) Adaptive behaviour			
No	61·3 (60·3 - 62·3)	70·8 (69·8 - 71·7)	15·2 (13·1 - 17·3)
Yes	38·7 (37·7 - 39·7)	29·2 (28·3 - 30·2)	84·8 (82·7 - 86·9)

Abbreviations: CI, confidence interval; FASD, fetal alcohol spectrum disorders; N, sample size. Note: Some percentages may not add to 100% due to rounding.

a Estimates pooled across imputation sets. N varies for each imputation set.

b FASD status based on the ‘Mid CNS/Any PAE’ screening algorithm.

c By definition all participants who meet criteria for FASD must have prenatal alcohol exposure.

d Participants who reported ‘none’ for alcohol consumption using this weekly dose/frequency measure may still have reported PAE on other measures of alcohol consumption (such as binge drinking, unit-based measures or continuation of pre-pregnancy drinking patterns).

e By definition all participants who meet criteria for FASD must have CNS impairment in ≥ 3 domains.

## 4 Discussion

The screen prevalence of FASD in this UK population-based sample was 6.0% using singly imputed data, 7.2% in complete case analysis, and 17.0% using multiply imputed data. The prevalence estimates, based on the complete case and single imputation strategies, are broadly consistent with the upper limits of other European studies, which have produced FASD prevalence estimates in the region of 1% to 5% (Lange et al., 2017a; Roozen et al., 2016). Although these estimates have some face validity, missing data patterns indicated that they were likely to be biased, as data were not missing completely at random. Participants with incomplete data experienced more adverse prenatal exposures and had poorer developmental outcomes relevant to FASD. Therefore, analyses with complete case and singly imputed data were likely to underestimate FASD prevalence. The single imputation method that we adopted (which assumed that missing data indicated no prenatal alcohol exposure and no impairment) is just one approach to single imputation that has been used in FASD prevalence studies. Other methods, for example where the imputed values could depend on other observed variables, may have produced higher estimates of FASD prevalence, but would have underestimated standard error.

The FASD screening prevalence estimate of 17.0%, based on multiply imputed data, may be a more robust estimate, due to the ability of this method to reduce bias due to missing data (Sterne et al., 2009). This estimate is significantly higher than existing estimates from active case ascertainment studies of FASD prevalence in Europe and the USA, which report a maximum prevalence of 10% (Lange et al., 2017a; Roozen et al., 2016; May et al., 2018), but lower than estimates from South Africa, where FASD prevalence is up to 28% (May et al., 2017). The UK has one of the highest levels of PAE in the world and, therefore, it is plausible that FASD prevalence would be relatively high. The pooled prevalence estimate for prenatal alcohol use is 15% in the USA, compared to 41% in the UK (Popova et al., 2017). Recent prospective studies produce higher estimates, suggesting that, consistent with results from this study, up to 79% of women in the UK drink while pregnant, with 33% at binge levels (Nykjaer et al., 2014; O'Keeffe et al., 2015).

### 4.1 FASD subtypes

ARND was the most common subtype of FASD, accounting for 15.4% of screen positive cases in analyses with multiply imputed data. The screen prevalence of ARND in this sample is higher than existing European estimates, while pFAS and FAS prevalence is lower. European studies have produced estimates of up to 0.8% for ARND, 1.7% for FAS and 5.0% for pFAS (May et al., 2006; May et al., 2011; Petkovic & Barisic, 2010; Petkovic & Barisic, 2013; Okulicz-Kozaryn et al., 2017). Simulation methods, based on PAE data, suggest that 0.6% of children in the UK may have FAS (Popova et al., 2017). Therefore, our combined prevalence estimate of 1.6% for pFAS/FAS may represent an underestimate. Facial scan data were collected at age 15 and evidence suggests that the FAS facial features become less prominent over time (Spohr et al., 1994). This may have led to reduced detection of pFAS/FAS in this study, but will not have influenced total FASD prevalence estimates.

The higher prevalence of ARND that we report, relative to the existing literature, may be explained partly by differences in study design. Many existing active case ascertainment studies of FASD follow a tiered screening protocol based on child dysmorphology, with brief neurobehavioural measures (May et al., 2006; May et al., 2011; Okulicz-Kozaryn et al., 2017) and, consequently, ARND is likely to be “severely undercounted” (May et al., 2011 (p. 2346)). Assessments of child phenotype in the ALSPAC dataset are more extensive than those that have been possible in active case ascertainment studies and this too is likely to have contributed to higher prevalence estimates for FASD, relative to existing studies. Furthermore, there is no universally accepted diagnostic framework for assessing FASD. Although there is broad consensus on FASD subtypes and the core features, diagnostic frameworks differ in the specific criteria, thresholds and nomenclature used to define FASD. This leads to variations in FASD classifications and subsequent prevalence estimates throughout the literature (Coles et al., 2016).

### 4.2 Strengths and limitations

To the best of our knowledge, this is the first study to estimate FASD screening prevalence in a UK-based general population sample. It provides a novel approach to FASD case ascertainment for epidemiological studies, including the use of multiple imputation methods to reduce the bias and imprecision introduced by missing data; the development and application of new screening algorithms for FASD; and the validation of these algorithms using blind expert panel review.

The study design had the following advantages. It is likely to increase capture of the full spectrum of FASD, since it did not rely on dysmorphology screening as a gateway to recruitment; it facilitated a large population-based investigation of FASD using a comprehensive range of measures to assess child phenotype in a manner that was significantly less costly and resource intensive than traditional active case ascertainment methods; and, as it used existing data, it could be conducted without additional consent, which maximised participation rates. Therefore, given that it has not yet been possible to conduct an active case ascertainment study of FASD in the UK, the method described in this study arguably provided the best currently available means of exploring the epidemiology of FASD at the population level.

However, there are important limitations. Classifications by the screening algorithms are not equivalent to a formal FASD diagnosis. An ideal clinical assessment for FASD would include a specialised in-person evaluation with relevant assessments completed at the same time, including genetic microarray testing to support differential diagnosis. Given the opportunity for a gold standard clinical assessment, it is possible that some of the children would not be considered to have FASD. That said, since self-reported prenatal alcohol use is likely to be underreported, some children with FASD may have not been identified by the screening algorithms.

Other limitations stem from the concept of FASD as a whole. The only feature of FASD that is specific to PAE is the facial phenotype. To date, a unique neurobehavioural profile for FASD has not been determined (Lange et al., 2017b). As the FASD Canadian guidelines for diagnosis note, “the face of FAS is the result of a specific effect of ethanol teratogenesis altering growth of the midface and brain. Those exposed to other embryotoxic agents may display a similar, but not identical, phenotypic facial development, impaired growth, a higher frequency of anomalies and developmental and behavioural abnormalities… Knowledge of exposure history will decrease the possibility of misdiagnosing FASD.” (Chudley et al., 2005 (pS7)). While we incorporated expert clinical judgement in our algorithm specification and validation process, it was not feasible to conduct individualised assessments of FASD. Therefore, in the simplest terms, the screening prevalence estimates reported in this study indicate that at least 6% of children were exposed to alcohol prenatally and had evidence of significant CNS impairment. It is not possible to prove conclusively that PAE was the key causal factor in determining the outcomes of these children. Equally, it is not possible to rule out alcohol as an important causal factor.

The validity of the prevalence estimates necessarily depend on the validity of the screening algorithms. Specificity estimates indicated that the ‘Mid CNS/Any PAE’ primary screening algorithm may have overestimated FASD, due to a high proportion of false positive results. Our selection of an algorithm that required evidence of any level of PAE as sufficient for consideration for FASD is consistent with the views of the expert validation panel, current antenatal guidelines, which recommend abstinence from alcohol as the safest option during pregnancy, and with evidence that suggests that there is no known safe level of PAE. Multiple co-occurring risk factors and maternal characteristics influence blood alcohol concentrations, the duration of fetal alcohol exposure and, therefore, alcohol teratogenicity. This has led some to question whether it will ever be possible to determine a ‘safe’ threshold for PAE (Clarren & Cook, 2016). Nevertheless, we recognise that it is unlikely that all children with CNS impairment and any level of alcohol exposure in pregnancy will have FASD through causative mechanisms.

The apparently low specificity values may also be due to an imperfect reference standard. Qualitative data, reported elsewhere (McQuire, 2018), suggested that many of the profiles that were classified as ‘not FASD’ by the panel were considered possible cases, subject to further investigation. Therefore, it seemed reasonable to favour high sensitivity, rather than high specificity, when choosing which of the algorithms to use for the screening prevalence analysis. The fact that the complete case and single imputation prevalence estimates were similar to those from existing active case ascertainment studies that have used these missing data strategies offers further support for the validity of the screening algorithms. Furthermore, the screen positive prevalence of FASD remained relatively high (12.7%–12.8%) in sensitivity analyses that applied two FASD screening algorithms with lower sensitivity and higher specificity values to the data. Nevertheless, our validation sample was relatively small (N = 31) due to practical constraints and further algorithm validation studies are warranted.

Although ALSPAC benefits from repeated prospective measurement of many prenatal exposures, a fundamental limitation of observational studies of prenatal exposures is the risk of measurement bias due to the use of self-report methods, in the absence of reliable biomarkers for objective measurements (McQuire et al., 2016).

PAE data were collected between 1991 and 1992, when there were no formal UK guidelines for drinking in pregnancy. Despite changes in guidance, patterns of prenatal alcohol consumption in ALSPAC are similar to recently published estimates (Nykjaer et al., 2014; O'Keeffe et al., 2015), suggesting that results may reflect present day patterns of PAE and, therefore, FASD. Although, because FASD is determined by a complex interplay of multiple factors that co-occur with maternal alcohol use, FASD prevalence could be subject to change based on the relative prevalence of risk and protective factors.

Mothers in the ALSPAC sample were slightly more affluent and children had higher levels of educational achievement than the general population, which poses further limitations on the ability to generalise findings from this sample to the general population of the UK (Boyd et al., 2013; Fraser et al., 2013). Specifically, the estimates of FASD prevalence in this sample may be lower than estimates derived from samples with lower socioeconomic status and those that include children with poorer educational outcomes on average.

## 5 Conclusions

FASD is potentially a common cause of developmental disability in the UK that is under ascertained. Active case ascertainment studies of FASD are urgently needed to clarify the current epidemiology of FASD in the general population of the UK.

## Conflicts of interest

None.

## Contributors' statement

Dr McQuire conceived of the study design and led the development of the screening algorithms, analysis and wrote and revised the manuscript.

Professor Paranjothy, Dr Hurt and Professor Kemp contributed to the study design, interpretation and revised the manuscript.

Dr Mukherjee, Mrs. Higgins and Professor Kemp contributed to the development of the screening algorithms and were members of the case conference validation panel.

Drs Greene and Farewell advised on the statistical aspects of this study and revised the manuscript.

All authors contributed to data interpretation and approved the final manuscript.

## Appendix A Supplementary data

Supplementary material Image 1
