# Supplementary material for: Screening prevalence of fetal alcohol spectrum disorders in a region of the United Kingdom: A population-based birth-cohort study
Source: Prev Med. 2019 Jan;118:344–51. doi: 10.1016/j.ypmed.2018.10.013 (PMC6344226; doi:10.1016/j.ypmed.2018.10.013)
Supplement: Supplementary file 1 — Supplementary material [file mmc1.docx]

**Supplementary Appendix**

**Supplement to: McQuire, C, Mukherjee, R, Hurt, L, Higgins, A, Greene, G, Farewell, D, Kemp, A, Paranjothy, S. Screening prevalence of fetal alcohol spectrum disorders in a region of the United Kingdom: a population-based birth-cohort study.**

# Appendix 1: FASD screening algorithm specifications and measures

Supplemental Table 1a: Schematic representation of the screening algorithms for FASD that were generated by varying combinations of the central nervous system (CNS) and prenatal alcohol exposure (PAE) criteria. Full definitions for the Liberal, Mid, Strict and Revised CNS criteria, and the terminology for all PAE categories are provided in Supplemental Table 1b.


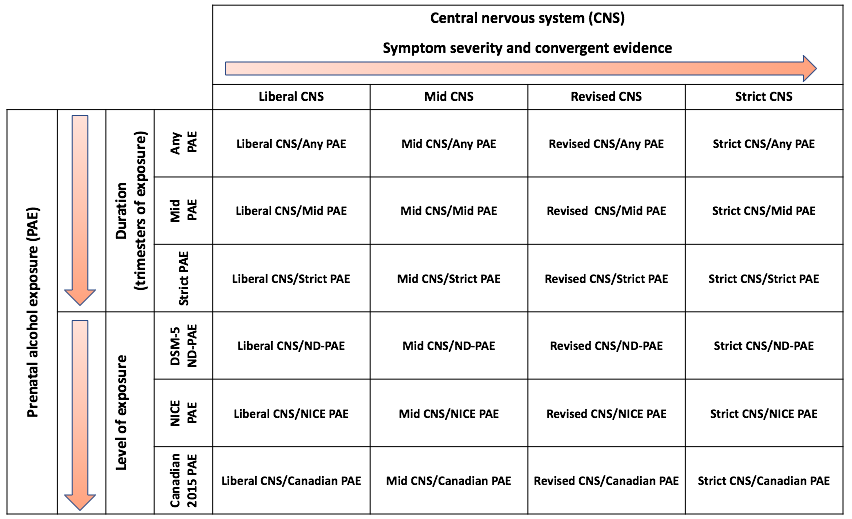


Supplemental Table 1b: FASD screening algorithm specifications (continued overleaf). Further details of the assessment measures are presented in Supplemental Table 1c.

| Domain | Subdomain | Case-definition specification | | | |
| --- | --- | --- | --- | --- | --- |
|  |  | Liberal CNS | Mid CNS | Strict CNS | Revised CNS |
| 1. Central Nervous System   (CNS)^a^  Impairment in ≥ 3 subdomains | ***a) Hard and soft neurologic signs*** | ≤5^th^ percentile  in ALSPAC coordination test **OR** ≥ 2 seizures not due to postnatal insult **OR** cerebral palsy | ≤5^th^ percentile in ALSPAC coordination test **OR** cerebral palsy | As for Mid | ≤5^th^ percentile  in ALSPAC coordination test **OR** ≥ 2 seizures not due to postnatal insult) **OR** cerebral palsy |
|  |  |  |  |  |  |
|  |  |  |  |  |  |
|  | ***b) Brain structure*** | Head circumference ≤ 2nd percentile at birth | Head circumference ≤ 2nd percentile at birth **AND** age 7 | As for Mid | Head circumference ≤ 2nd percentile at birth **OR** age 7 |
|  | ***c) Cognition*** | Score ≤ 70 on total, verbal or performance IQ **OR** discrepancy of ≥ 1 SD between subdomains | As for Liberal | As for Liberal | Score ≤ 70 on total, verbal or performance IQ **OR** discrepancy of ≥ 1 SD between subdomains. Not impaired if discrepancy between subdomains but IQ ≥ 120 |
|  | ***d) Communication: receptive and expressive*** | Score ≤ 2 SD from the mean for WOLD listening comprehension **OR** WOLD expressive language **OR** teacher-reported communication impairment at any time point | Impaired on any two of the measures in this domain | Score ≤ 2 SD from the mean for WOLD listening comprehension **AND** WOLD expressive language **AND** teacher-reported impairment at any time point | Impaired on any two of the measures in this domain (including consideration of SEN communication needs) |
|  |  |  |  |  |  |
|  |  |  |  |  |  |
|  | ***e) Academic achievement*** | Failing to meet the expected level at school at any time point (Key Stage 1 Level 1 or W; Key Stage 2 < Level 4) **OR** SEN at any time point | Failing to meet the expected level at school at all time points (Key Stage 1 Level 1 or W; Key Stage 2 < Level 4) **OR** SEN at any time point | SEN at any time point | Failing to meet the expected level at school at all time points **OR** SEN at any time point. Do not mark as impaired if IQ ≤ 79. Do not mark as impaired if normal educational attainment and a reason other than cognitive and learning needs for SEN. |
|  |  |  |  |  |  |
|  |  |  |  |  |  |
|  | ***f) Memory*** | Score ≤3^rd^ percentile on Forward Digit Span **OR** Non-Word Repetition Task | As for Liberal | ≤3^rd^ percentile on Forward Digit Span | Score ≤3^rd^ percentile on Forward Digit Span **OR** Non-Word Repetition Task |
|  |  |  |  |  |  |
|  | ***g) Executive functioning and abstract reasoning*** | Score ≤3^rd^ percentile for any of the available measures: Opposite Worlds task, Counting Span task, Stop Signal task, Backwards Digit Span task | Score ≤3^rd^ percentile for two of the available measures | Score ≤3^rd^ percentile for  Counting Span **AND** Backwards Digit Span tasks  **OR** Score ≤3^rd^ percentile for Stop-Signal **AND** Opposite Worlds tasks | Score ≤3^rd^ percentile for two of the available measures |
|  |  |  |  |  |  |
|  |  |  |  |  |  |
|  |  |  |  |  |  |
|  | ***h) Attention deficit/hyperactivity*** | Score ≤3^rd^ percentile for Sky Search **OR** ADHD **OR** high SDQ hyperactivity | ADHD **OR** high SDQ hyperactivity | ADHD | Convergent evidence across 2 measures or impairment reported by both informants for SDQ (teacher and parent) **OR** ADHD |
|  |  |  |  |  |  |
|  |  |  |  |  |  |
|  | ***i) Adaptive behaviour, social skills, social communication*** | High SDQ peer **OR** conduct problems **OR** ODD/CD **OR** ≥ 7 DANVA errors **OR** score ≥ 9 on SCDC **OR** autism **OR** teacher-reported emotional or behavioural difficulties | High SDQ peer **OR** conduct problems **OR** ODD/CD **OR** autism **OR** two of the following: ≥ 7 DANVA errors **OR** score ≥ 9 on SCDC **OR** teacher-reported emotional or behavioural difficulties | ODD/CD **OR** autism | Impaired on any two of the measures in this domain or impairment reported by both informants for SDQ (teacher and parent) including consideration of SEN behavioural, emotional and social development needs **OR** autism |
| 1. Growth |  | ≤ 9^th^ percentile for: birth weight **AND/OR** birth length and postnatal height **AND/OR** birth and postnatal BMI | | | |
| 1. Face |  | Full facial phenotype (for FAS): short palpebral fissure length (≤ 2·5^th^ percentile) **AND** smooth philtrum **AND** thin upper lip (equivalent to ranks 4-5 on the lip-philtrum guide)  Partial facial phenotype (for pFAS): any two features from the full facial phenotype | | | |
| 1. Prenatal alcohol exposure (PAE) |  | **Any PAE:** Any level of prenatal alcohol exposure at any time in pregnancy; **Mid PAE:** Two trimesters of prenatal alcohol exposure and/or binge drinking; **Strict PAE:** Three trimesters of prenatal alcohol exposure and/or binge drinking; **Canadian PAE**: Seven or more standard Canadian drinks per week (11·9 UK units) or any binge drinking^b^; **ND-PAE**: > 13 drinks per month, with > 2 drinks per occasion; **NICE PAE**: ≥ 1-2 drinks once or twice per week or ≥ 4 units of alcohol^d^ | | | |
| Abbreviations: CNS, central nervous system; DANVA, Diagnostic Analysis of Non-Verbal Accuracy; IQ, intelligence quotient; ND-PAE, Neurodevelopmental Disorder-Prenatal Alcohol Exposure; NICE, National Institute for Health and Care Excellence; ODD/CD, oppositional/conduct disorder; PAE, prenatal alcohol exposure; SD, standard deviation; SCDC, Social Communication Disorders Checklist; SDQ, Strengths and Difficulties Questionnaire; SEN, special educational needs; WOLD, Weschler Objective Language Dimensions.  ^a^ CNS criterion met if there is evidence of impairment in ≥ 3 subdomains (a - i). The Revised CNS case-definition requires that this includes impairment in the subdomains that measure adaptive functioning (e and i).  ^b^ The FASD Canadian 2016 guidelines suggest that more than one binge drinking episode is required to meet the PAE criteria; however, the ALSPAC data categorise binge drinking as: none, 1-2 days, 3-4 days, 5-10 days, > 10 days, or every day per month. Therefore, it was not possible to separate participants with one binge drinking episode from those with two or more.  ^c^ DSM-5 ND-PAE criterion of ‘more than minimal exposure’. Exposed to alcohol at any time during gestation, including prior to pregnancy recognition, and the exposure level was more than minimal (i.e. more than 13 drinks in any one month, with more than two drinks on any drinking occasion.  ^d^ Drinking in excess of NICE 2008 antenatal guideline limits ≥1-2 drinks once or twice per week; equivalent to ~ 32g / 4 units of alcohol. | | | | | |

Supplemental Table 1c: Overview of the assessment measures used in one or more of the FASD screening algorithms (continued overleaf)

| Criterion | Domain | ALSPAC variable | Age assessed  (years) | Suggested threshold for impairment | Method for deriving threshold for impairment | Test details |
| --- | --- | --- | --- | --- | --- | --- |
| Growth  (A) | Weight | Weight | Birth - 9 | ≤ 9^th^ percentile at birth | Standard norms | Excel LMS Growth Add-in based on UK growth norms.^1,2^  Centiles for pre-term babies < 35 weeks gestation were generated using the Fenton Growth Calculator.^3^ |
|  | Height | Height | Birth - 9 | ≤ 9^th^ percentile at birth and postnatally (up to puberty) | Standard norms |  |
|  | Body mass index (BMI) | BMI | Birth - 9 | ≤ 9^th^ percentile at birth and postnatally (up to puberty) | Standard norms |  |
| Face  (B) | Palpebral fissure length | 3D facial scan data | 15.5 | ≤ 2.5^th^ percentile | Standard norms | FAS Diagnostic and Prevention Network Z-Score Calculator for palpebral fissure length.^4,5^ |
|  | Thin upper lip |  |  | Equivalent to 4/5 on the lip-philtrum guide | Standard norms | Wilson Scale for Lips.^6^ |
|  | Smooth philtrum |  |  |  | Standard norms |  |
| CNS  (C) | 1. Hard and soft neurologic signs (including sensory-motor) | Movement score | 7 | Top 5% (95^th^ percentile)  Score: ≥ 6 for girls  ≥ 7 for boys | Research literature | ALSPAC coordination test (modified version of Movement Assessment Battery)  The Movement Assessment Battery for Children was used to test the children’s motor ability.^7^  It comprises three sections, assessing static and dynamic balance, manual dexterity and ball skills.  Because of time constraints, it was not possible to conduct the whole assessment, so specific subtests from each of the three sections were carried out:  Manual dexterity: placing pegs and threading lace  Ball skills: bean bags  Balance: heel to toe walking  Odd et al. derived a summary score based on the three tasks. 'The top (i.e. indicating worse performance) 5^th^ centile of this summed score was used to define severe motor coordination difficulties as has been used previously in the literature.^8^ |
|  |  | Seizures | 1 - 13 | > 1 seizure not due to fever, breath-holding or response to immunisation (i.e. due to epilepsy) | Expert opinion and FASD Canadian 2005 guidelines | ALSPAC asks the child’s caregiver:  Has the child ever had a seizure, fit or convulsion?  And whether the seizure was due to factors including immunisation, fever or breath-holding, epilepsy. |
|  |  | Cerebral palsy | NR | Cerebral palsy | Expert opinion | Cerebral palsy reported by mother/carer |
|  | 1. Brain structure | Head circumference | Birth and 7 | ≤ 2nd percentile | Standard norms | Excel LMS Growth Add-in based on UK growth norms.^1,2^ |
|  | 1. Cognition | WISC-III  (short-form) | 8 | Score ≤ 70 on total, verbal or performance IQ  Discrepancy of at least 1 SD (i.e. 15 points) between the subdomains (i.e. verbal and performance IQ). | Standard norms and FASD Canadian 2005 guidelines | WISC-III (short form)  Alternate WISC items were used for all subtests, except for the coding subtest which was administered in its full form.^9-12^  Administered by members of the psychology team.  Raw scores were calculated according to the items used in the alternate item form of the WISC, making the raw scores comparable to those that would have been obtained had the full test been administered.^13,14^ |
|  | 1. Communication: receptive and expressive | WOLD (Wechsler Objective Language Dimensions) | 8 | Score ≤3 for wold_list  Score ≤3 for wold_express | Based on the distribution of ALSPAC participant data. | Wechsler Objective Language Dimensions (WOLD; modified):^14^  Listening comprehension: The listening comprehension subtest of the WOLD is divided into two parts. The first is a single word receptive vocabulary test, similar to the vocabulary subtest of the WISC. This was not therefore used.  In the second part of the assessment, the child listens to the tester read aloud a paragraph about a picture, which the child is shown. The child then answers questions on what they have heard.  The child has to make inferences about what was read to them and answer the questions verbally. The task was discontinued if the child got three consecutive questions incorrect. Alternate items from the standard test were sampled except where the item had American cultural loading. In those cases, the next item was selected.  Expressive language: The WOLD has two expressive language subtests. In the second subtest three tasks were performed. Firstly, a picture was shown to the child who was asked to describe the scene, as if to someone who was not present and so could not see the picture. Secondly, the child was shown a map and asked to give directions from one location to another, using the shortest route possible and finally they were asked to explain the steps involved in a sequential task of putting batteries into a torch using pictures to help.  These tasks assess the child’s descriptive, narrative and sequencing skills. All responses in this task were recorded on audio tape for later coding on five features, relating to the relevance, accuracy and logicality of the child’s responses. In the full WOLD assessment, each task has two examples. Only one of each was used in the ALPSAC tests. |
|  |  | Communication (general) | 7 and 10 | ‘Yes’ indicates reported impairment at any time point | Expert opinion | Teacher-reported speech and language difficulties in school (needing special assistance) |
|  | 1. Academic achievement | Special needs | 9 to 10  10 to 11  11 to 12 | 2 (School Action) 3 (School Action plus) and 4 (SEN statement) indicate impairment | Standard norms | Pupil Level Annual School Census (PLASC) recorded SEN: School action, school action plus and statement.^15^ |
|  |  | Academic attainment | 6-7 | “Failing to meet expected level”  Key stage 1: Level 1 or W | Standard norms | \| **Key Stage** \| **Range of levels within which most children will work** \| **Target that most children reach by the end of the key stage** \| **Further information about Key Stage levels**  **(Source: *ALSPAC SATS Doc* and *ALSPAC Key Stage 2 File)*** \| \| --- \| --- \| --- \| --- \| \| 1 \| 1 - 3 \| 2 \| W = Code W (“working towards level 1”) means that the child was assessed but didn’t achieve level 1. \| \| 2 \| 2 - 5 \| 4 \| The basic scale consists of levels 1, 2, 3 and 4+, with grades A, B and C within level 2.  Point Score - All Subjects  4+ = 27  3 = 21  2A = 17  2B = 15  2C = 13  1 = 9 \| |
|  |  |  | 10-11 | “Failing to meet expected level”  Key Stage 2: < Level 4 | Standard norms |  |
|  | 1. Memory | Short term memory | 8 | Score ≤ 3 | Based on the distribution of ALSPAC participant data. | WISC-III forward digit span^13,14,16,17^  Children repeated lists of digits in order. |
|  |  |  | 8 | Score ≤ 2 | Based on the distribution of ALSPAC participant data. | Modified Non-word Repetition Test^14,18^  Twelve nonsense words, four each of 3, 4 and 5 syllables and conforming to English rules for sound combinations.  The child was asked to listen to each word via an audio cassette recorder and then repeat each item. |
|  | 1. Executive functioning and abstract reasoning | Working memory | 10 | Score ≤ 2 | Based on the distribution of ALSPAC participant data. | Counting Span Task^19-21^  The child was presented with red and blue dots on a white screen. The child was asked to point to and count the number of red dots out loud (the processing component).  The children were shown:  • Two practice sets of two screens  • Three sets of two screens  • Three sets of three screens  • Three sets of four screens  • Three sets of five screens  After each set, the child was asked to recall the number of red dots seen on each screen  in the order they were presented within that set (the storage component). |
|  |  | Working memory | 8 | ≤ 2 correct responses | Based on the distribution of ALSPAC participant data. | WISC-III (short form) Backwards Digit Span.^13,14,16,17^  Children repeated lists of digits in reverse order. |
|  |  | Inhibition | 10 | ≤ 12 correct responses | Based on the distribution of ALSPAC participant data. | Stop-signal paradigm^20-22^  This task observes the child’s ability to inhibit a body movement that has already been requested using a computerized measure of impulsivity.  When a ‘stop signal cue’ (bleep) was not heard the child was asked to press the corresponding button according to what was presented on screen. When the bleep was sounded the child was told to refrain from pressing the response button, therefore  inhibiting the stimulus response. |
|  |  | Opposite Worlds Task | 8 | Time (secs)  ≥ 28 for males aged 7 - <9  ≥ 24.5 for males aged 9 - 11  ≥ 26 for females aged 7 - <9  ≥ 24 for females aged 9 - 11 | Based on the distribution of ALSPAC participant data. | TEACh- Opposite Worlds Task^14,23^  A Stroop task, where the child is required to give a verbal response that contradicts the visual information he or she is given. The child is shown a trail made up of the numbers 1 and 2. In the ‘opposite world’ condition, the child must call out ‘two’ when he or she reaches a 1 and ‘one’ when he or she reaches a 2. |
|  | 1. Attention deficit/hyperactivity | Selective attention | 8 | ≥ 11 for males aged 7 - <9  ≥ 9 for males aged 9 - 11  ≥ 8 for females aged 7 - 11 | Based on the distribution of ALSPAC participant data. | TEA-Ch Sky Search Task^14,23^  The child was asked to circle identical pairs of spaceships as quickly as possible but not missing any out. The child was asked to tick a box on the sheet to indicate that he/she had circled all the identical pairs he/she could find. |
|  |  | DAWBA ADHD | 7.5 | DAWBA ADHD | Clinical diagnosis by ALSPAC team | Development and Well-Being Assessment (DAWBA)^24^  DAWBA diagnoses were classified by psychiatrists.  For ADHD and oppositional/conduct disorders, the diagnostic procedure considers the teacher report in addition to the parent report. Full DSM-IV diagnoses were only made for children for whom the parent report had been completed. |
|  |  | SDQ Hyperactivity Score | 7 to 11 years | High SDQ  Score ≥ 8 | Standard norms | Strengths and difficulties questionnaire (SDQ)^25^ |
|  | 1. Adaptive behaviour, social skills, social communication | SDQ Peer Problems Score |  | High SDQ  Score ≥ 5 for teacher-rated  Score ≥ 4 for parent-rated | Standard norms |  |
|  |  | SDQ Conduct Problems Score |  | High SDQ  Score ≥ 4 | Standard norms |  |
|  |  | DAWBA Oppositional-conduct disorder | 7.5 | DAWBA Oppositional-conduct disorder | Clinical diagnosis by ALSPAC team | Development and Well-Being Assessment (DAWBA)^24^  DAWBA diagnoses were classified by psychiatrists.  For ADHD and oppositional/conduct disorders, the diagnostic procedure considers the teacher report in addition to the parent report.  Full DSM-IV diagnoses were only made for children for whom the parent report had been completed. |
|  |  | Diagnostic Analysis of Non-Verbal Accuracy (DANVA) | 8 | ≥ 7 errors | Research literature | Diagnostic Analysis of Non-Verbal Accuracy (DANVA)^26^  The DANVA faces subtest comprises 24 photos of child faces, with each face showing one of four emotions: happiness, sadness, anger or fear. The photos are presented to the child for two seconds each and he or she must respond as to whether the person in the photo is happy, sad, angry or afraid. |
|  |  | Social cognition | 7.5 and 11 | Score ≥ 9 | Standard norms | Social Communication Disorders Checklist (SCDC)^27-29^  Measure of social-cognitive dysfunction. |
|  |  | Autism spectrum disorder | Up to age 11 | Any autism spectrum disorder | Clinical diagnosis recorded in NHS or PLASC | Autism identified by NHS or PLASC records.^30^ |
|  |  | Teacher reported emotional or behavioural difficulties | 7 and 10 | ‘Yes’ indicates reported impairment at any time point | Expert opinion | Teacher-reported emotional or behavioural difficulties at school |
| Prenatal alcohol exposure  (D) | Prenatal alcohol exposure (PAE) | Prenatal alcohol exposure | Prenatal (reported for each trimester):  Data collected at approximately 8, 18 and 32 weeks gestation and 8 weeks postpartum. | N/A | Self-reported alcohol consumption during pregnancy including information about dose, frequency and timing.  **Timing**: First, second or third trimester  **Duration**: None, one trimester, two trimesters, all trimesters  **Dose/frequency**: <1 glass per week  At least one glass per week  1-2 glasses daily  3-9 glasses daily  >9 glasses daily  The ALSPAC questionnaire defined a glass of alcohol as equivalent to a pub measure of spirits, ½ pint lager/beer, wine glass of wine etc.  **‘Binge’ drinking in the last month:** 1-2 days  3-4 days  5-10 days  >10 days  everyday  ‘Binge drinking’ defined as the consumption of 2 pints of beer, 4 glasses of wine, 4 pub measures of spirits or equivalent on a single occasion. Information on first trimester binge drinking was not available.  **Dose (units):** UK standard units | |

# Appendix 2: Case-conference sampling strategy for validation of the FASD screening algorithms

In the validation stage of this study, we selected a stratified random sample of 31 participant profiles from the eligible ALSPAC sample to be considered by an expert case conference panel. The sample was stratified to ensure that at least two participant profiles were considered for each of the 24 algorithm specifications and that at least three participants did not meet criteria for FASD under any of the case ascertainment algorithms. By definition, participants who met criteria for the more stringent FASD classifications (i.e. higher levels/duration of PAE or more convergent evidence or severe symptoms for the CNS criteria) also met criteria for the less stringent classifications. Supplemental Figure 2a presents the number of participants in the validation sample who met criteria for each of the case ascertainment algorithms.

*Supplemental Figure 2a: Sample for the FASD case ascertainment algorithm validation process. The number in each cell corresponds to the number of participants who met criteria for FASD under the corresponding algorithm specification. Total N = 31.*

#
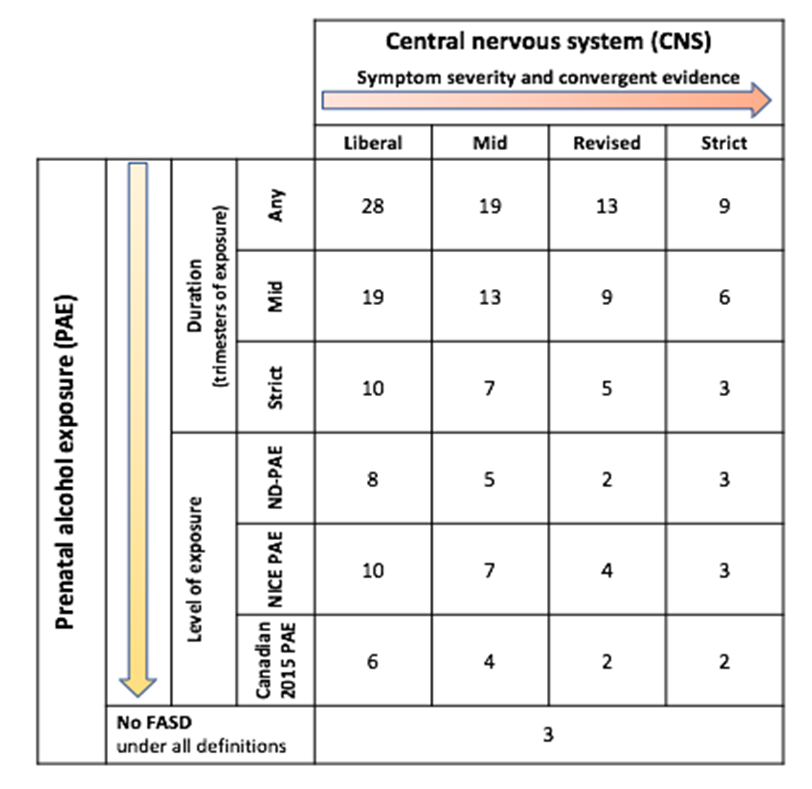


# Appendix 3: Comparison of participant characteristics by missing data strategy

To investigate which factors were most influential in accounting for the higher prevalence of FASD in the analysis with multiple imputed data, we compared the patterns of PAE and clinical characteristics across each of the missing data strategies. The relative pattern of sociodemographic, PAE and clinical characteristics of individuals with and without FASD was broadly consistent across analyses. Compared to the complete case and single imputation methods, the multiply imputed data contained a higher proportion of individuals with PAE (up to 10% increase), growth deficiency (up to 4% increase) and CNS impairment in at least three subdomains (up to 13% increase) (see Supplemental Figure 3a).

Supplemental Figure 3a: Proportion of participants who met criteria for each of the main FASD domains in analyses with complete case, single imputation, and multiple imputation methods

Supplemental Figure 3b presents a comparison of the proportion of participants with impairment in each CNS subdomain, by missing data strategy. For all CNS subdomains, the multiply imputed data had the highest proportion of participants with impairment. The largest increases, relative to the complete case and singly imputed data, were in the cognitive (up to 10% increase) and adaptive functioning (up to 12% increase) subdomains. Overall, the increased prevalence of impairment across CNS subdomains, combined with the increased prevalence of PAE accounted for the higher prevalence of FASD in the analyses with multiply imputed data, compared to the complete case and single imputation methods.

Supplemental Figure 3b: Proportion of participants who met criteria for impairment in each CNS subdomain in analyses with complete case, single imputation and multiple imputation methods

#

# Appendix 4: Multiple imputation method and missing data frequencies

To increase the plausibility of the missing at random (MAR) assumption, we used an inclusive strategy for the imputation model, which included hypothesised risk factors for FASD, sociodemographic variables, clinical characteristics and auxiliary variables. Models that impute subcomponents of composite outcomes have been shown to lead to increased precision and reduced bias, relative to methods that impute the composite outcomes directly.^31,32^ Therefore, we imputed the missing values of FASD subcomponents (including psychological test scores and PAE measures), and then combined these subcomponent values following imputation, to construct the final FASD status and FASD subtype composite outcomes. We used multiple imputation by chained equations (MICE) in Stata 14.2.^33,34^ We used 10 cycles to produce each imputed data set and generated 20 imputation sets. Imputation models were specified as binary (logit), ordered categorical (ologit), non-ordered categorical (mlogit) and continuous (regress) for each variable as appropriate. The imputation model specification and missing data frequencies are presented in Supplemental Table 4a.

Supplemental Table 4a: Multiple imputation model specification and missing data frequencies (continued overleaf)

|  |  | N eligible = 13,495 | | |  |
| --- | --- | --- | --- | --- | --- |
| Variable description | Categories | N available | Missing (%) | | Imputation command |
| Alcohol dose/frequency during pregnancy | None/<1 glass per week/1-6 glasses per week/7+ glasses per week | 12,947 | | 4 | ologit |
| Binge drinking during pregnancy (Questionnaire B) | No/Yes | 12,519 | | 7 | logit |
| Binge drinking during pregnancy (Questionnaire C) | No/Yes | 8,411 | | 38 | logit |
| Alcohol consumption pre-pregnancy | Never/<1 glass per wk/1+ glasses per week/1-2 glasses daily/3-9 glasses daily/10+ glasses daily | 12,602 | | 7 | ologit |
| Alcohol drinking change during pregnancy | No evidence of continued drinking/Had more or No change | 11,621 | | 14 | logit |
| Measures of alcohol per week (Questionnaire A) | Continuous | 11,375 | | 16 | regress |
| Measures of alcohol per week (Questionnaire C) | Continuous | 6,619 | | 51 | regress |
| Short palpebral fissure length | No/Yes | 4,370 | | 68 | logit |
| Philtrum shape | Smooth/Indentation near nose/Indentation in middle/Indentation near vermillion border/Deep groove from nose to vermillion border/Deep groove to Cupid's bow | 4,370 | | 68 | ologit |
| Upper lip fullness | Thin/Medium/Thick | 4,370 | | 68 | ologit |
| Low coordination | No/Yes | 6,520 | | 52 | logit |
| More than one seizure not due to postnatal insult | No/Yes | 11,646 | | 14 | logit |
| Small head circumference (OFC) at birth | No/Yes | 8,340 | | 38 | logit |
| Small head circumference (OFC) at age 7 | No/Yes | 7,449 | | 45 | logit |
| Verbal IQ | Continuous | 6,830 | | 49 | regress |
| Performance IQ | Continuous | 6,821 | | 49 | regress |
| Full-scale IQ | Continuous | 6,800 | | 50 | regress |
| Low listening comprehension | No/Yes | 6,821 | | 49 | logit |
| Low expressive language | No/Yes | 4,493 | | 67 | logit |
| Communication problems at school | No/Yes | 4,100 | | 70 | logit |
| Special educational needs | No/Yes | 11,312 | | 16 | logit |
| Low academic attainment at KS1 and KS2 | No/Yes | 11,824 | | 12 | logit |
| Low non-word repetition | No/Yes | 6,809 | | 50 | logit |
| Low forward digit span | No/Yes | 6,702 | | 50 | logit |
| Low opposite world task | No/Yes | 6,657 | | 51 | logit |
| Low counting span | No/Yes | 6,455 | | 52 | logit |
| Low stop signal | No/Yes | 6,426 | | 52 | logit |
| Low backwards digit span | No/Yes | 6,683 | | 50 | logit |
| ADHD | No/Yes | 7,952 | | 41 | logit |
| SDQ hyperactivity (parent-rated) | No/Yes | 9,402 | | 30 | logit |
| SDQ hyperactivity (teacher-rated) | No/Yes | 9,145 | | 32 | logit |
| Low selective attention | No/Yes | 6,638 | | 51 | logit |
| Oppositional-conduct disorder | No/Yes | 7,952 | | 41 | logit |
| SDQ peer problems (parent-rated) | No/Yes | 9,414 | | 30 | logit |
| SDQ peer problems (teacher-rated) | No/Yes | 9,145 | | 32 | logit |
| SDQ conduct problems (parent-rated) | No/Yes | 9,412 | | 30 | logit |
| SDQ conduct problems (teacher-rated) | No/Yes | 9,137 | | 32 | logit |
| Significant social cognitive dysfunction | No/Yes | 8,678 | | 36 | logit |
| Emotional/behavioural problems at school | No/Yes | 9,088 | | 33 | logit |
| Autism | No/Yes | 13,495 | | 0 | - |
| DANVA ≥ 7 errors | No/Yes | 6,304 | | 53 | logit |
| Growth impairment | No/Yes | 13,419 | | 1 | logit |
| Illicit drug use during pregnancy | No/Yes | 12,886 | | 5 | logit |
| Smoking during pregnancy | No/Yes | 13,304 | | 1 | logit |
| Multivitamin supplements during pregnancy | No/Yes | 12,898 | | 4 | logit |
| Iron supplements during pregnancy | No/Yes | 12,907 | | 4 | logit |
| Zinc supplements during pregnancy | No/Yes | 12,906 | | 4 | logit |
| Calcium supplements during pregnancy | No/Yes | 12,905 | | 4 | logit |
| Folic acid supplements during pregnancy | No/Yes | 12,903 | | 4 | logit |
| Any vitamin supplements during pregnancy | No/Yes | 12,908 | | 4 | logit |
| Daily calorie intake during pregnancy | Continuous | 11,660 | | 14 | regress |
| Retinol (RNI met) | No/Yes | 11,660 | | 14 | logit |
| Calcium(RNI met) | No/Yes | 11,660 | | 14 | logit |
| Omega-3 (RNI met) | No/Yes | 11,660 | | 14 | logit |
| Folate (RNI met) | No/Yes | 11,660 | | 14 | logit |
| Riboflavin (RNI met) | No/Yes | 11,660 | | 14 | logit |
| Selenium (RNI met) | No/Yes | 11,660 | | 14 | logit |
| Vitamin B12 (RNI met) | No/Yes | 11,660 | | 14 | logit |
| Vitamin C (RNI met) | No/Yes | 11,660 | | 14 | logit |
| Vitamin E (RNI met) | No/Yes | 11,660 | | 14 | logit |
| Zinc (RNI met) | No/Yes | 11,660 | | 14 | logit |
| Iodine (RNI met) | No/Yes | 11,660 | | 14 | logit |
| Iron (RNI met) | No/Yes | 11,660 | | 14 | logit |
| Magnesium (RNI met) | No/Yes | 11,660 | | 14 | logit |
| Niacin (RNI met) | No/Yes | 11,660 | | 14 | logit |
| Phosphorous (RNI met) | No/Yes | 11,660 | | 14 | logit |
| Potassium (RNI met) | No/Yes | 11,660 | | 14 | logit |
| Sodium RNI met) | No/Yes | 11,660 | | 14 | logit |
| Thiamin (RNI met) | No/Yes | 11,660 | | 14 | logit |
| Vitamin B6 (RNI met) | No/Yes | 11,660 | | 14 | logit |
| Marital status | Married/Not married | 12,646 | | 6 | logit |
| Home ownership | Mortgaged or owned/Council or housing assoc./Rented (private)/Other | 12,595 | | 7 | mlogit |
| Maternal social class | Professional/Managerial or technical/Skilled non-manual/Skilled manual/Partly skilled/unskilled | 9,718 | | 28 | ologit |
| Paternal social class | Professional/Managerial or technical/Skilled non-manual/Skilled manual/Partly skilled/unskilled | 10,590 | | 22 | ologit |
| Highest educational qualification (maternal) | CSE/Vocational/O Level/A Level/Degree | 11,988 | | 11 | ologit |
| Highest educational qualification (paternal) | CSE/Vocational/O Level/A Level/Degree | 11,516 | | 15 | ologit |
| Maternal age (years) | < 20/20-29/30+ | 13,495 | | 0 | - |
| Religion (maternal) | None/Christian/Other | 11,783 | | 13 | mlogit |
| Unplanned pregnancy | No/Yes | 12,620 | | 6 | logit |
| Total life events during pregnancy | Continuous | 10,204 | | 24 | regress |
| Weighted total life events during pregnancy | Continuous | 10,204 | | 24 | regress |
| Relationship problems during pregnancy | No/Yes | 12,801 | | 5 | logit |
| Very affected by relationship problems during pregnancy | No/Yes | 12,801 | | 5 | logit |
| Bereavement during pregnancy (any) | No/Yes | 12,781 | | 5 | logit |
| Very affected by bereavement during pregnancy | No/Yes | 12,781 | | 5 | logit |
| Major financial problem during pregnancy | No/Yes | 12,763 | | 5 | logit |
| Very affected by major financial problems during pregnancy | No/Yes | 12,763 | | 5 | logit |
| Moved house during pregnancy | No/Yes | 12,984 | | 4 | logit |
| Very affected by house move during pregnancy | No/Yes | 12,769 | | 5 | logit |
| Very affected by illness or accident during pregnancy | No/Yes | 12,783 | | 5 | logit |
| Illness or accident during pregnancy | No/Yes | 12,783 | | 5 | logit |
| Partner ill during pregnancy | No/Yes | 12,765 | | 5 | logit |
| Very affected by partner illness during pregnancy | No/Yes | 12,765 | | 5 | logit |
| Child ill during pregnancy | No/Yes | 12,762 | | 5 | logit |
| Very affected by child illness during pregnancy | No/Yes | 12,762 | | 5 | logit |
| Depression during pregnancy | No/Yes | 12,551 | | 7 | logit |
| Anxiety during pregnancy | No/Yes | 12,469 | | 8 | logit |
| Social support during pregnancy | Continuous | 11,050 | | 18 | regress |
| Any abuse (mother) | No/Yes | 12,902 | | 4 | logit |
| Maternal grandmother had alcoholism | No or Don't Know/Yes | 11,716 | | 13 | logit |
| Maternal rs1229984 genotype (>= 1 rare allele) | No/Yes | 7,712 | | 43 | logit |
| Maternal impulsivity | No/Yes | 7,149 | | 47 | logit |
| Perinatal trauma/complications | No/Yes | 12,665 | | 6 | logit |
| Postnatal binge drinking (8 weeks) | No/Yes | 8,214 | | 39 | logit |
| Postnatal alcohol problems | No/Yes | 9,680 | | 28 | logit |
| Parity | 0/1/2/>2 | 12,487 | | 7 | ologit |
| Gestational age at delivery | Continuous | 13,495 | | 0 | - |
| Previous miscarriage | 0/1/≥2 | 12,539 | | 7 | ologit |
| Ultrasound at any point in pregnancy | No/Yes | 10,858 | | 20 | logit |
| Maternal BMI (pre-pregnancy) | Underweight/Normal/Overweight/Obese | 11,140 | | 17 | ologit |
| Child gender | Male/Female | 13,495 | | 0 | - |
| Maternal ethnicity | White/Non-White | 11,904 | | 12 | logit |

# Appendix 5: Characteristics of participants according to data completeness

Supplemental Table 5a: Comparison of the distribution of sociodemographic factors, prenatal exposures and FASD outcome variables among participants with complete data, compared to those who had missing data for one or more of the measures required to ascertain FASD status (continued overleaf)

|  | Eligible sample  N = 13,495  N (%)^a^ | | Participants with complete data  N = 223  (N [%]) | | Participants with missing data for one or more of the measures required to ascertain FASD  status  N = 13,272  (N [%]) | |
| --- | --- | --- | --- | --- | --- | --- |
| SOCIODEMOGRAPHIC FACTORS | | | | | | |
| Maternal age (years) | | | | | | |
| <29 | 8,480 (62·9) | | 105 (47·1) | | 8,375 (63·1) | |
| 30+ | 5,015 (37·2) | | 118 (52·9) | | 4,897 (36·9) | |
| Maternal ethnicity | | | | | | |
| White | 11,600 (97·5) | | Censored^b^ | | | |
| Non-White | 304 (2·6) | |  |  |  |  |
| Marital status | | | | | | |
| Not married | 3,163 (25·0) | | 28 (12·6) | | 3,135 (25·3) | |
| Married | 9,483 (75·0) | | 194 (87·4) | | 9,289 (74·7) | |
| Maternal social class | | | | | | |
| Professional | 573 (5·9) | | 12 (5·7) | | 561 (5·9) | |
| Managerial/technical | 3,060 (31·5) | | 74 (35·4) | | 2,986 (31·4) | |
| Skilled non-manual | 4,153 (42·7) | | 96 (45·9) | | 4,057 (42·7) | |
| Skilled manual | 763 (7·9) | | 13 (6·2) | | 750 (7·9) | |
| Partly skilled/unskilled | 1,169 (12·0) | | 14 (6·7) | | 1,155 (12·2) | |
| Paternal social class | | | | | | |
| Professional | 1,160 (11·0) | | 40 (18·5) | | 1,120 (10·8) | |
| Managerial/technical | 3,596 (34·0) | | 74 (34·3) | | 3,522 (34·0) | |
| Skilled non-manual | 1,156 (10·9) | | 33 (15·3) | | 1,123 (10·8) | |
| Skilled manual | 3,333 (31·5) | | 53 (24·5) | | 3,280 (31·6) | |
| Partly skilled/unskilled | 1,345 (12·7) | | 16 (7·4) | | 1,329 (12·8) | |
| Maternal education | | | | | | |
| CSE | 2,416 (20·2) | | 19 (8·5) | | 2,397 (20·4) | |
| Vocational | 1,182 (9·9) | | 13 (5·8) | | 1,169 (9·9) | |
| O Level | 4,149 (34·6) | | 77 (34·5) | | 4,072 (34·6) | |
| A Level | 2,706 (22·6) | | 76 (34·1) | | 2,630 (22·4) | |
| Degree | 1,535 (12·8) | | 38 (17·0) | | 1,497 (12·7) | |
| Paternal education | | | | | | |
| CSE | 3,013 (26·2) | | 25 (11·3) | | 2,988 (26·5) | |
| Vocational | 975 (8·5) | | 21 (9·5) | | 954 (8·5) | |
| O Level | 2,436 (21·2) | | 49 (22·2) | | 2,387 (21·1) | |
| A Level | 3,004 (26·1) | | 68 (30·8) | | 2,936 (26·0) | |
| Degree | 2,088 (18·1) | | 58 (26·2) | | 2,030 (18·0) | |
| Home ownership status |  |  | |  | |  |
| Mortgaged/owned | 9,240 (73·4) | | 205 (92·3) | | 9,035 (73·0) | |
| Council/housing association | 2,016 (16·0) | | 5 (2·3) | | 2,011 (16·3) | |
| Rented (private) | 902 (7·2) | | 5 (2·3) | | 897 (7·3) | |
| Other | 437 (3·5) | | 7 (3·2) | | 430 (3·5) | |
| PRENATAL EXPOSURES | | | | | | |
| Prenatal alcohol use (any) |  | |  | |  | |
| No | 4,132 (31·1) | | 61 (27·4) | | 4,071 (31·2) | |
| Yes | 9,135 (68·9) | | 162 (72·7) | | 8,973 (68·8) | |
| Prenatal alcohol exposure (max dose/frequency during pregnancy) | | | | | | |
| None | 4,171 (32·2) | | 63 (28·3) | | 4,108 (32·3) | |
| <1 glass per week | 5,480 (42·3) | | 103 (46·2) | | 5,377 (42·3) | |
| 1-6 glasses per week | 2,872 (22·2) | | 49 (22·0) | | 2,823 (22·2) | |
| 7+ glasses per week | 424 (3·3) | | 8 (3·6) | | 416 (3·3) | |
| Prenatal binge drinking | | | | | | |
| No | 9,927 (77·8) | | 180 (80·7) | | 9,747 (77·7) | |
| Yes | 2,839 (22·2) | | 43 (19·3) | | 2,796 (22·3) | |
| Alcohol use before pregnancy | | | | | | |
| None | 1,048 (8·3) | | 5 (2·2) | | 1,043 (8·4) | |
| ≤ 1 - 6 glasses per week | 10,144 (80·5) | | 191 (85·7) | | 9,953 (80·4) | |
| 7+ glasses per week | 1,410 (11·2) | | 27 (12·1) | | 1,383 (11·2) | |
| Prenatal smoking | | | | | | |
| No | 9,601 (72·2) | | 192 (86·1) | | 9,409 (71·9) | |
| Yes | 3,703 (27·8) | | 31 (13·9) | | 3,672 (28·1) | |
| Prenatal illicit drug use | | | | | | |
| No | 12,464 (96·7) | | Censored^b^ | | | |
| Yes | 422 (3·3) | |  |  |  |  |
| Prenatal vitamin supplement use (any) | | | | | | |
| No | 5,938 (46·0) | | 95 (42·6) | | 5,843 (46·1) | |
| Yes | 6,970 (54·0) | | 128 (57·4) | | 6,842 (53·9) | |
| Prenatal stressful life events | | | | | | |
| Mean (SD) | 7 (4) | | 6 (4) | | 7 (4) | |
| Social support score | | | | | | |
| Mean (SD) | 20 (5) | | 21 (4) | | 20 (5) | |
| Prenatal anxiety | | | | | | |
| No | 9,545 (76·6) | | 184 (82·5) | | 9,361 (76·4) | |
| Yes | 2,924 (23·4) | | 39 (17·5) | | 2,885 (23·6) | |
| Prenatal depression | | | | | | |
| No | 9,940 (79·2) | | 194 (87·0) | | 9,746 (79·1) | |
| Yes | 2,611 (20·8) | | 29 (13·0) | | 2,582 (20·9) | |
| Unplanned pregnancy |  |  | |  | |  |
| No | 8,722 (69·1) | | 187 (83·9) | | 8,535 (68·9) | |
| Yes | 3,898 (30·9) | | 36 (16·1) | | 3,862 (31·2) | |
| CLINICAL CHARACTERISTICS | | | | | | |
| Facial phenotype |  |  | |  | |  |
| Short palpebral fissure length | | | | | | |
| No | 3,390 (77·6) | | 174 (78·0) | | 3,216 (77·6) | |
| Yes | 980 (22·4) | | 49 (22·0) | | 931 (22·5) | |
| Smooth philtrum | | | | | | |
| No | 3,461 (79·2) | | 166 (74·4) | | 3,295 (79·5) | |
| Yes | 909 (20·8) | | 57 (25·6) | | 852 (20·5) | |
| Thin upper lip | | | | | | |
| No | 3,951 (90·4) | | 193 (86·6) | | 3,758 (90·6) | |
| Yes | 419 (9·6) | | 30 (13·5) | | 389 (9·4) | |
| Central nervous system |  |  | |  | |  |
| Coordination test | | | | | | |
| Normal | 6,204 (95·1) | | 218 (97·8) | | 5,986 (95·1) | |
| Poor motor coordination | 316 (4·9) | | 5 (2·2) | | 311 (4·9) | |
| Head circumference at birth | | | | | | |
| Normal | 8,229 (98·7) | | Censored^b^ | | | |
| Small (<2^nd^ percentile) | 111 (1·3) | |  |  |  |  |
| Head circumference at age 7 | | | | | | |
| Normal | 6,841 (91·8) | | 200 (89·7) | | 6,641 (91·9) | |
| Small (<2^nd^ percentile) | 608 (8·2) | | 23 (10·3) | | 585 (8·1) | |
| Full scale IQ | | | | | | |
| Mean (SD) | 104 (17) | | 109 (15) | | 104 (17) | |
| Verbal IQ | | | | | | |
| Mean (SD) | 107 (17) | | 111 (15) | | 107 (17) | |
| Performance IQ | | | | | | |
| Mean (SD) | 100 (17) | | 102 (17) | | 100 (17) | |
| Significant difference between IQ subdomains | | | | | | |
| No | 3,772 (55·5) | | 119 (53·4) | | 3,653 (55·5) | |
| Yes | 3,028 (44·5) | | 104 (46·6) | | 2,924 (44·5) | |
| WOLD listening comprehension task | | | | | | |
| Normal | 6,627 (97·2) | | 216 (96·9) | | 6,411 (97·2) | |
| Low performance | 194 (2·8) | | 7 (3·1) | | 187 (2·8) | |
| WOLD expressive language |  |  | |  | |  |
| Normal | 4,324 (96·2) | | Censored^b^ | | | |
| Low performance | 169 (3·8) | |  |  |  |  |
| Speech and language problems at school | | | | | | |
| No | 3,552 (86·6) | | 217 (97·3) | | 3,335 (86·0) | |
| Yes | 548 (13·4) | | 6 (2·7) | | 542 (14·0) | |
| Special educational needs |  |  | |  | |  |
| No | 8,867 (78·4) | | 195 (87·4) | | 8,672 (78·2) | |
| Yes | 2,445 (21·6) | | 28 (12·6) | | 2,417 (21·8) | |
| Low academic attainment at Key Stage 1 and 2 | | | | | | |
| No | 10,174 (86·1) | | 209 (93·7) | | 9,965 (85·9) | |
| Yes | 1,650 (14·0) | | 14 (6·3) | | 1,636 (14·1) | |
| Non-word repetition task | | | | | | |
| Normal | 6,565 (96·4) | | 212 (95·1) | | 6,353 (96·5) | |
| Low performance | 244 (3·6) | | 11 (4·9) | | 233 (3·5) | |
| Forward digit span task | | | | | | |
| Normal | 6,557 (97·8) | | 214 (96·0) | | 6,343 (97·9) | |
| Low performance | 145 (2·2) | | 9 (4·0) | | 136 (2·1) | |
| Opposite worlds task | | | | | | |
| Normal | 6,460 (97·0) | | Censored^b^ | | | |
| Low performance | 197 (3·0) | |  |  |  |  |
| Counting span task | | | | | | |
| Normal | 6,166 (95·5) | | 218 (97·8) | | 5,948 (95·4) | |
| Low performance | 290 (4·5) | | 5 (2·2) | | 284 (4·6) | |
| Stop signal task | | | | | | |
| Normal | 6,214 (96·7) | | 215 (96·4) | | 5,999 (96·7) | |
| Low performance | 212 (3·3) | | 8 (3·6) | | 204 (3·3) | |
| Backwards digit span task | | | | | | |
| Normal | 6,182 (92·5) | | 204 (91·5) | | 5,978 (92·5) | |
| Low performance | 501 (7·5) | | 19 (8·5) | | 482 (7·5) | |
| ADHD | | | | | | |
| No | 7,786 (97·9) | | 215 (96·4) | | 7,571 (98·0) | |
| Yes | 166 (2·1) | | 8 (3·6) | | 158 (2·0) | |
| SDQ hyperactivity | | | | | | |
| No | 10,174 (86·4) | | 195 (87·4) | | 9,979 (86·4) | |
| Yes | 1,604 (13·7) | | 28 (12·6) | | 1,576 (13·6) | |
| Oppositional-conduct disorder | | | | | | |
| No | 7,703 (96·9) | | 215 (96·4) | | 7,488 (96·9) | |
| Yes | 249 (3·1) | | 8 (3·6) | | 241 (3·1) | |
| SDQ peer problems | | | | | | |
| No | 9,594 (81·4) | | 186 (83·4) | | 9,408 (81·4) | |
| Yes | 2,189 (18·6) | | 37 (16·6) | | 2,152 (18·6) | |
| SDQ conduct problems | | | | | | |
| No | 9,530 (80·9) | | 189 (84·8) | | 9,341 (80·8) | |
| Yes | 2,249 (19·1) | | 34 (15·3) | | 2,215 (19·2) | |
| SCDC social communication problems | | | | | | |
| No | 7,820 (90·1) | | 206 (92·4) | | 7,614 (90·1) | |
| Yes | 858 (9·9) | | 17 (7·6) | | 841 (10·0) | |
| Emotional and/or behavioural problems at school | | | | | | |
| No | 8,245 (90·7) | | 210 (94·2) | | 8,035 (90·6) | |
| Yes | 843 (9·3) | | 13 (5·8) | | 830 (9·4) | |
| Autism | | | | | | |
| No | 13,414 (99·4) | | Censored^b^ | | | |
| Yes | 81 (0·6) | |  |  |  |  |
| DANVA task | | | | | | |
| Normal | 4,891 (77·6) | | 177 (79·4) | | 4,714 (77·5) | |
| Low performance | 1,413 (22·4) | | 46 (20·6) | | 1,367 (22·5) | |
| Growth |  |  | |  | |  |
| Growth deficiency | | | | | | |
| No | 12,310 (91·7) | | 214 (96·0) | | 12,096 (91·7) | |
| Yes | 1,109 (8·3) | | 9 (4·0) | | 1,100 (8·3) | |
| AUXILIARY VARIABLES |  |  | |  | |  |
| Pregnancy/perinatal complications | | | | | | |
| No | 8,881 (70·1) | | 157 (70·7) | | 8,724 (70·1) | |
| Yes | 3,784 (29·9) | | 65 (29·3) | | 3,719 (29·9) | |
| Binge drinking (8 weeks postpartum) | | | | | | |
| No | 4,973 (60·5) | | 152 (69·1) | | 4,821 (60·3) | |
| Yes | 3,241 (39·5) | | 68 (30·9) | | 3,173 (39·7) | |
| Postnatal alcohol problems (maternal self-report and AUDIT 5 to 18 years postpartum) | | | | | | |
| No | 7,900 (81·6) | | 162 (72·7) | | 7,738 (81·8) | |
| Yes | 1,780 (18·4) | | 61 (27·4) | | 1,719 (18·2) | |
| Parity |  |  | |  | |  |
| 0 | 5,597 (44·8) | | 109 (49·8) | | 5,488 (44·7) | |
| 1 | 4,369 (35·0) | | 72 (32·9) | | 4,297 (35·0) | |
| 2 | 1,781 (14·3) | | 26 (11·9) | | 1,755 (14·3) | |
| >2 | 740 (5·9) | | 12 (5·5) | | 728 (5·9) | |
| Gestational age at delivery (weeks) | | | | | | |
| Mean (SD) | 39·5 (1·8) | | 39·5 (1·5) | | 39·5 (1·9) | |
| Previous miscarriage |  |  | |  | |  |
| 0 | 9,861 (78·6) | | 169 (76·5) | | 9,692 (78·7) | |
| 1 | 2,017 (16·1) | | 45 (20·4) | | 1,972 (16·0) | |
| ≥2 | 661 (5·3) | | 7 (3·2) | | 654 (5·3) | |
| Ultrasound scan during pregnancy | | | | | | |
| No | 533 (4·9) | | 9 (4·0) | | 524 (4·9) | |
| Yes | 10,035 (95·1) | | 214 (96·0) | | 10,111 (95·1) | |
| Maternal BMI (pre-pregnancy) |  |  | |  | |  |
| Underweight | 558 (5·0) | | 9 (4·2) | | 549 (5·0) | |
| Normal | 8,280 (74·3) | | 162 (75·4) | | 8,118 (74·3) | |
| Overweight | 1,684 (15·1) | | 33 (15·4) | | 1,651 (15·1) | |
| Obese | 618 (5·6) | | 11 (5·1) | | 607 (5·6) | |
| Child sex |  |  | |  | |  |
| Female | 6,541 (48·5) | | 101 (45·3) | | 6,853 (51·6) | |
| Male | 6,954 (51·5) | | 122 (54·7) | | 6,419 (48·4) | |
| ^a^ Sample size for each variable differs from eligible sample due to missing data. Percentages do not always sum to 100 due to rounding.  ^b^ Data from these cells are not reported due to a cell count < 5 for one of the strata.  Abbreviations: BMI, body mass index; CSE, Certificate of secondary education; DANVA, Diagnostic Analysis of Nonverbal Accuracy; SCDC, Social Communication Disorder Checklist; SD, standard deviation; SDQ, Strengths and Difficulties Questionnaire; WOLD, Weschler Objective Language Dimensions. | | | | | | |

# Appendix 6: FASD screening algorithm performance

Supplemental Table 6a: Test accuracy statistics for ALSPAC FASD screening algorithms (index test), compared to the case-conference panel FASD classification (reference standard). Measure with shortest 0,1 distance highlighted in bold (lower 0,1 values indicate better performance). This is the algorithm that was selected for the primary prevalence analyses.

| FASD case-definition | TP | FP | FN | TN | N^a^ | Sens %  (95% CI) | Spec %  (95% CI) | 0,1 | Included in prevalence analysis |
| --- | --- | --- | --- | --- | --- | --- | --- | --- | --- |
| Liberal CNS; Any PAE | 11 | 17 | 0 | 3 | 31 | 100 (74 - 100) | 15 (5 - 36) | 0·85 | No |
| Liberal CNS; Mid PAE | 8 | 11 | 3 | 9 | 31 | 73 (43 - 90) | 45 (26 - 66) | 0·61 | No |
| Liberal CNS; Strict PAE | 4 | 6 | 6 | 14 | 30 | 40 (17 - 69) | 70 (48 - 85) | 0·67 | No |
| Liberal CNS; Canadian PAE | 2 | 4 | 9 | 16 | 31 | 18 (5 - 48) | 80 (56 - 94) | 0·84 | No |
| Liberal CNS; ND-PAE | 4 | 4 | 7 | 16 | 31 | 36 (15 - 65) | 80 (56 - 94) | 0·67 | No |
| Liberal CNS; NICE PAE | 5 | 5 | 6 | 15 | 31 | 45 (21 - 72) | 75 (51 - 91) | 0·60 | No |
| Mid CNS; Any PAE | **10** | **9** | **1** | **11** | **31** | **91 (62 - 98)** | **55 (34 - 74)** | **0·46** | **Yes - primary analysis** |
| Mid CNS; Mid PAE | 7 | 6 | 4 | 14 | 31 | 64 (35 - 85) | 70 (48 - 85) | 0·47 | Yes - sensitivity analysis |
| Mid CNS; Strict PAE | 4 | 3 | 6 | 17 | 30 | 40 (17 - 69) | 85 (64 - 95) | 0·62 | No |
| Mid CNS; Canadian PAE | 2 | 2 | 9 | 18 | 31 | 18 (5 - 48) | 90 (68 - 99) | 0·82 | No |
| Mid CNS; ND-PAE | 3 | 2 | 8 | 18 | 31 | 27 (9 - 57) | 90 (68 - 99) | 0·73 | No |
| Mid CNS; NICE PAE | 4 | 3 | 7 | 17 | 31 | 36 (15 - 65) | 85 (64 - 95) | 0·65 | No |
| Strict CNS; Any PAE | 6 | 3 | 5 | 13 | 27 | 55 (28 - 79) | 81 (57 - 93) | 0·49 | No |
| Strict CNS; Mid PAE | 5 | 1 | 6 | 15 | 27 | 45 (21 - 72) | 94 (72 - 99) | 0·55 | No |
| Strict CNS; Strict PAE | 3 | 0 | 7 | 16 | 26 | 30 (11 - 60) | 100 (81 - 100) | 0·70 | No |
| Strict CNS; Canadian PAE | 2 | 0 | 9 | 16 | 27 | 18 (5 - 48) | 100 (81 - 100) | 0·82 | No |
| Strict CNS; ND-PAE | 3 | 0 | 8 | 16 | 27 | 27 (10 - 57) | 100 (81 -100) | 0·73 | No |
| Strict CNS; NICE PAE | 3 | 0 | 8 | 16 | 27 | 27 (10 - 57) | 100 (81 - 100) | 0·73 | No |
| Revised CNS; Any PAE | 7 | 6 | 4 | 14 | 31 | 64 (35 - 85) | 70 (48 - 85) | 0·47 | Yes - sensitivity analysis |
| Revised CNS; Mid PAE | 6 | 3 | 5 | 17 | 31 | 55 (28 - 79) | 85 (64 - 95) | 0·48 | No |
| Revised CNS; Strict PAE | 3 | 2 | 7 | 18 | 30 | 30 (11 - 60) | 90 (70 - 97) | 0·71 | No |
| Revised CNS; Canadian PAE | 1 | 1 | 10 | 19 | 31 | 9 (2 - 38) | 95 (76 - 99) | 0·91 | No |
| Revised CNS; ND-PAE | 1 | 1 | 10 | 19 | 31 | 9 (2 - 38) | 95 (76 - 99) | 0·91 | No |
| Revised CNS; NICE PAE | 2 | 2 | 9 | 18 | 31 | 18 (5 - 48) | 90 (70 - 97) | 0·82 | No |
| Abbreviations: CI, confidence interval; CNS, central nervous system; FN, false negative; FP, false positive; PAE, prenatal alcohol exposure; Sens, sensitivity; Spec, specificity; TN, true negative; TP, true positive.  Notes: ^a^ N refers to number of participants out of the total case-conference sample (N = 31) with sufficient information available to determine FASD classification for each case-definition category. For example, some participants had missing data on PAE for one or more trimester and, therefore, had insufficient data available to meet Strict PAE case-ascertainment algorithms. 0,1 statistic indicates distance from the top left-hand corner of a Receiver Operating Characteristic (ROC) plot with lower values indicating better test performance, defined as d = √ ([1-Sensitivity]^2^ + [1-Specificity]^2^). | | | | | | | | | |

Supplemental Figure 6a: Receiver operating characteristic (ROC) plot depicting diagnostic accuracy of the FASD screening algorithms, relative to the FASD classifications assigned by the expert case-conference panel. Dashed arrows represent distance from the top left-hand corner of the ROC plot (which would indicate perfect agreement with the case-conference panel) and text boxes indicate the 0,1 statistic for the three FASD screening algorithms with the highest level of agreement with the case-conference panel. Note that the position of the Mid CNS/Mid PAE and Revised CNS/Any PAE algorithms overlap, as they had the same 0,1 value.


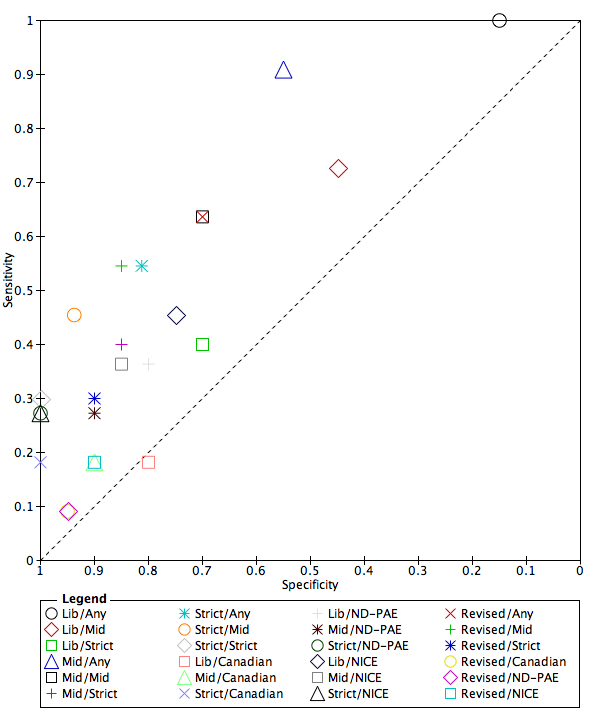


0.46

0.47

**References:**

1. *LMSgrowth* [computer program]. Tyne & Wear, UK: Harlow Printing, Ltd.; 2016.

2. Cole TJ, Freeman JV, Preece MA. British 1990 growth reference centiles for weight, height, body mass index and head circumference fitted by maximum penalized likelihood. *Stat Med.* 1998;**17**(4):407-429.

3. *Fenton Preterm Growth Charts: Research Bulk Calculator* [computer program]. Calgary, Alberta: University of Calgary; 2015.

4. *Palpebral fissure length z-score calculator* [computer program]. Washington. <https://depts.washington.edu/fasdpn/htmls/diagnostic-tools.htm>. Accessed May 22, 2018.

5. Strömland K, Chen Y, Norberg T, Wennerström K, Michael G. Reference values of facial features in Scandinavian children measured with a range-camera technique. *Scand J Plast Reconstr Surg Hand Surg.* 1999;33(1):59-65.

6. Wilson C, Playle R, Toma A, Zhurov A, Ness A, Richmond S. The prevalence of lip vermilion morphological traits in a 15-year-old population. *Am J Med Genet A.* 2013;161(1):4-12.

7. Henderson SE SD. *Movement Assessment Battery for Children manual.* Sidcup, UK: The Psychological Corporation; 1992.

8. Odd DE, Lingam R, Emond A, Whitelaw A. Movement outcomes of infants born moderate and late preterm. *Acta Paediatr.* 2013;102(9):876-882.

9. Donders J. A short form of the WISC-III for clinical use. *Psychol Assess.* 1997;9(1):15-20.

10. Donders J. Using a short form of the WISC-III: sinful or smart? *Child Neuropsychol.* 2001;**7**(2):99-103.

11. Donders J, Warschausky S. Validity of a short form of the WISC-III in children with traumatic head injury. *Child Neuropsychol.* 1996;2(3):227-232.

12. Finch AJ, Ollendick TH, Ginn FW. WISC short forms with mentally retarded children. *Am J Ment Defic.* 1973;78(2):144–149.

13. Wechsler D. *WISC III (Wechsler Intelligence Scale for Children).* London: The Psychological Corporation; 1992.

14. ALSPAC. Focus at 8: built files documentation. University of Bristol; 2006. http://www.bristol.ac.uk/alspac/researchers/access/. Accessed November 26, 2015.

15. ALSPAC. PLASC file: data collected from the Pupil Level Annual School Census. University of Bristol; 2008. http://www.bristol.ac.uk/alspac/researchers/access/. Accessed November 26, 2015.

16. Gerton BK, Brown TT, Meyer-Lindenberg A, et al. Shared and distinct neurophysiological components of the digits forward and backward tasks as revealed by functional neuroimaging. *Neuropsychologia.* 2004;42(13):1781-1787.

17. Hale JB, Hoeppner JAB, Fiorello CA. Analyzing digit span components for assessment of attention processes. *J Psychoeduc Assess.* 2002;20(2):128-143.

18. Gathercole SE, Willis CS, Baddeley AD, Emslie H. The children's test of nonword repetition: a test of phonological working memory. *Memory*. 1994;2(2):103-27.

19. Case R, Kurland DM, Goldberg J. Operational efficiency and the growth of short-term memory span. *J Exp Child Psychol.* 1982;33(3):386-404.

20. Handley SJ, Capon A, Beveridge M, Dennis I, Evans JSB. Working memory, inhibitory control and the development of children's reasoning. *Think Reason.* 2004;10(2):175-195.

21. ALSPAC. Focus at 10: built files documentation. University of Bristol; 2005. http://www.bristol.ac.uk/alspac/researchers/access/. Accessed November 26, 2015.

22. Logan GD, Cowan WB, Davis KA. On the ability to inhibit simple and choice reaction time responses: a model and a method. *J Exp Psychol Hum Percept Perform.* 1984;10(2):276-291.

23. Manly T, Robertson, I.H., Anderson, V., Nimmo-Smith, I. . *Test of Everyday Attention for Children (TEA-Ch).* Bury St. Edmonds: Thames Valley Test Company Limited; 1999.

24. Goodman R, Ford T, Richards H, Gatward R, Meltzer H. The Development and Well-Being Assessment: description and initial validation of an integrated assessment of child and adolescent psychopathology. *J Child Psychol Psychiatry.* 2000;41(5):645-655.

25. Goodman R. The Strengths and Difficulties Questionnaire: a research note. *J Child Psychol Psychiatry.* 1997;38(5):581-586.

26. Nowicki S, Duke MP. Nonverbal receptivity: The Diagnostic Analysis of Nonverbal Accuracy (DANVA). In: Hall JA & Bernieri FJ, editors. *Interpersonal Sensitivity: Theory and Measurement.* Mahwah, NJ: Lawrence Erlbaum Associates Publishers; 2001:183-198.

27. Skuse DH, James RS, Bishop DVM, et al. Evidence from Turner's syndrome of an imprinted X-linked locus affecting cognitive function. *Nature.* 1997;387(6634):705-708.

28. Skuse DH, Mandy W, Steer C, et al. Social communication competence and functional adaptation in a general population of children: preliminary evidence for sex-by-verbal IQ differential risk. J Am Acad Child Adolesc Psychiatry*.* 2009;48(2):128-137.

29. Skuse DH, Mandy WPL, Scourfield J. Measuring autistic traits: heritability, reliability and validity of the Social and Communication Disorders Checklist. *Br J Psychiatry.* 2005;187(6):568-572.

30. Williams E, Thomas K, Sidebotham H, Emond A. Prevalence and characteristics of autistic spectrum disorders in the ALSPAC cohort. *Dev Med Child Neurol.* 2008;50(9):672-677.

31. Eekhout I, de Vet HC, Twisk JW, Brand JP, de Boer MR, Heymans MW. Missing data in a multi-item instrument were best handled by multiple imputation at the item score level. *J Clin Epidemiol.* 2014;67(3):335-342.

32. Plumpton CO, Morris T, Hughes DA, White IR. Multiple imputation of multiple multi-item scales when a full imputation model is infeasible. *BMC Res Notes.* 2016;9:doi: 10.1186/s13104-13016-11853-13105.

33. Royston P. Multiple imputation of missing values: further update of ice, with an emphasis on categorical variables. *Stata Journal.* 2009;9:466-477.

34. *Stata Statistical Software: Release 14* [computer program]. Texas: College Station, StataCorp LP; 2015.
